# Distinctive Surface Glycosylation Patterns Associated With Mouse and Human CD4^+^ Regulatory T Cells and Their Suppressive Function

**DOI:** 10.3389/fimmu.2017.00987

**Published:** 2017-08-21

**Authors:** Joana Cabral, Shirley A. Hanley, Jared Q. Gerlach, Neil O’Leary, Stephen Cunningham, Thomas Ritter, Rhodri Ceredig, Lokesh Joshi, Matthew D. Griffin

**Affiliations:** ^1^Regenerative Medicine Institute (REMEDI) at CÚRAM Centre for Research in Medical Devices, School of Medicine, College of Medicine, Nursing and Health Sciences, National University of Ireland, Galway, Ireland; ^2^Flow Cytometry Core Facility, National Centre for Biomedical Engineering Sciences (NCBES), National University of Ireland, Galway, Ireland; ^3^Glycoscience Group, National Centre for Biomedical Engineering Sciences (NCBES), National University of Ireland, Galway, Ireland; ^4^HRB Clinical Research Facility, National University of Ireland, Galway, Ireland

**Keywords:** regulatory T-cells, lectins, glycosylation, immune regulation, cellular immunology, T cell proliferation, Foxp3, *N*-glycan

## Abstract

Regulatory T-cells (Treg) are essential for maintaining immune homeostasis and tolerance. Surface glycosylation is ubiquitous on mammalian cells and regulates diverse biological processes. While it is currently well accepted that surface glycan expression influences multiple aspects of T-cell function, little is known about the relevance of glycosylation to Treg biology. This study aimed to profile the surface glycosylation characteristics of Treg in various lymphoid compartments of mouse and in human peripheral blood with comparison to non-regulatory, conventional CD4^+^ T-cells (Tconv). It also sought to determine the relationship between the surface glycosylation characteristics and suppressive potency of Treg. Lectin-based flow cytometric profiling demonstrated that Treg surface glycosylation differs significantly from that of Tconv in the resting state and is further modified by activation stimuli. In mouse, the surface glycosylation profiles of FoxP3^+^ Treg from spleen and lymph nodes were closely comparable but greater variability was observed for Treg in thymus, bone marrow, and blood. Surface levels of tri/tetra-antennary *N-*glycans correlated with expression of proteins known to be involved in Treg suppressive functions, including GITR, PD-1, PD-L1, CD73, CTLA-4, and ICOS. In coculture experiments involving purified Treg subpopulations and CD4^+^ or CD8^+^ Tconv, higher surface tri/tetra-antennary *N-*glycans was associated with greater Treg suppressive potency. Enzymatic manipulation of mouse Treg surface glycosylation resulting in a temporary reduction of surface *N*-glycans significantly reduced Treg capacity to suppress Tconv activation through contact-dependent mechanisms. Overall, these findings demonstrate that Treg have distinctive surface glycan characteristics that show variability across anatomical locations and are modulated by activation events. They also provide evidence of an important role for surface glycosylation in determining Treg phenotype and suppressive potency. These insights may prove relevant to the analysis of Treg in disease settings and to the further development of Treg-based immunotherapies.

## Introduction

Regulatory T-cells (Treg) can be distinguished from conventional CD4^+^ T-cells (Tconv) by intracellular expression of the transcription factor FoxP3 (1) and, in humans, by a CD25^high^CD127^low^ phenotype (2). Through multiple specialized functions that suppress the activities of other effector immune cells, Treg prevent autoimmunity and excessive immune responses to infection and tissue injury (3–6). Recently, investigation of the spectrum of Treg phenotypic and functional heterogeneity has revealed significant diversity in regard to their origin, development, activation status, migratory properties, and functional mechanisms (5, 7–13).

Posttranslational attachment of carbohydrates to proteins *via* glycosylation pathways profoundly influences their functional properties by modulating inter- and intra-molecular steric effects and by generating ligands for glycan-binding proteins (14). In mammals, effective immunity is dependent both on dynamic regulation of glycan attachments to proteins and on the expression of glycan-binding proteins (14, 15). Within the immune system, changes in glycosylation of cell surface and secreted molecules modulate self/non-self discrimination; leukocyte migration, homing, and apoptosis; B-cell receptor and T-cell receptor (TCR) activation; IgG Fc function; MHC-mediated antigen presentation; notch-dependent B- and T-cell development; and T-effector differentiation (14–16).

Although characterizations of T-cell glycosylation during development and activation have been reported (17–19), few such studies have evaluated Treg as a distinct T-cell subset. Furthermore, studies of Treg glycosylation have, thus far, focused on individual glycan structures as reflected in the binding of the *Amaranthus leucocarpus* lectin (20) and sialic acid-specific lectins (21) to human peripheral blood mononuclear cells (PBMCs) and of *Phaseolus vulgaris* leucoagglutinin (PHA-L) to mouse splenocytes (22). Here, we report the results of a detailed comparison of surface glycosylation characteristics of regulatory and conventional CD4^+^ T-cells and demonstrate a relationship between Treg glycan expression and suppressive potency.

## Materials and Methods

### Animals

C57BL/6 FoxP3.EGFP transgenic mice (23) were kindly provided by Dr. Karen English, Institute of Immunology, Maynooth University, Ireland. Experimental animals were housed and bred in a specific pathogen-free facility and were euthanized for blood and tissue collection at 5–12 weeks of age. All animal procedures were carried out under individual authorization from the Health Products Regulatory Authority of Ireland and the Environmental Protection Agency of Ireland and were approved by the NUI Galway Animal Care Research Ethics Committee.

### Immune Cell Isolation

Lymphocytes from primary and secondary lymphoid organs were isolated from C57BL/6 FoxP3.EGFP transgenic mice. Single cell suspensions from spleen, thymus, and lymph nodes were obtained by mechanical disruption. Bone marrow cells were obtained by flushing the femurs and tibiae using a 27-gauge (G) needle filled with culture medium [high-glucose DMEM (Gibco™, Carlsbad, CA, USA) supplemented with 10% FBS (Lonza, Basel, Switzerland), 1% penicillin/streptomycin (Gibco™), 1% l-glutamine (Gibco™), 1% HEPES solution (Sigma-Aldrich, St. Louis, MO, USA), 1% MEM non-essential amino acid solution (Sigma-Aldrich), and 50 µM 2-mercaptoethanol (Sigma-Aldrich)]. The resulting cell suspensions were individually filtered through a 40-µm Sefar petex mesh (Sefar, Heiden, Switzerland) to remove any debris and cellular aggregates. Erythrocytes were depleted from the cell suspensions by incubation with red blood cell (RBC) lysis buffer (eBioscience, San Diego, CA, USA) for 4 min at room temperature. Peripheral blood leukocytes (PBLs) were obtained from mouse blood collected immediately after euthanasia from the right ventricle, to which the RBC lysis buffer was applied twice. Human PBMCs were isolated from blood samples collected from healthy adult volunteers aged 24–64 years old by density gradient separation. Briefly, anticoagulated blood samples were gently placed on top of 4 ml of Ficoll-Paque Plus^®^ (GE Healthcare, Chalfont St. Giles, UK—or—Piscataway, NJ, USA) in a 15 ml tube and were centrifuged for 20 min at 1,250 RCF, 20°C, without acceleration or brake. Post-centrifugation, the various cellular constituents of the blood were separated in individual layers, with the PBMCs lying in the interface between the plasma and the Ficoll. The PBMCs were carefully collected from the interface using a 5 ml pipette and were washed with fluorescence-activated cell sorting (FACS) buffer [PBS, 2% FBS (Sigma-Aldrich), 0.05% NaN_2_ (Sigma-Aldrich)] then pelleted by centrifugation for 10 min at 512 RCF, 20°C. Collection of blood from healthy volunteers was performed by informed consent under a protocol approved by the Research Ethics Committee of the National University of Ireland, Galway.

### Flow Cytometry (FCM) and FACS

Single cell suspensions of freshly isolated or cultured mouse and human cells were washed and suspended in FACS buffer, incubated with various combinations of fluorochrome-conjugated antibodies for 30 min at 4°C, washed and re-suspended in FACS buffer prior to flow cytometric analysis. Murine cells from spleen, lymph node, and peripheral blood were labeled with anti-mouse CD4 antibody whereas thymic T-cells were additionally labeled with anti-mouse CD8α and bone marrow T-cells were identified by the concomitant expression of CD4 and TCR-β. Intracellular expression of the transcription factor FoxP3, indicated by GFP expression, was used to identify the Treg populations. Human T-cells were identified by labeling the isolated PBMCs with antibodies against CD4, CD25, and CD127 such that the CD4^+^CD127^low^CD25^high^ subset corresponded to the Treg population and the remainder of the CD4^+^ cells were considered to be the Tconv population. Phenotypic characterization was performed by incubating the cells with appropriate fluorochrome-conjugated antibodies (Table S3 in Supplementary Material; eBioscience) for 30 min, at 4°C. For the analysis of the expression of CTLA-4 and Helios, surface marker labeling was followed by intracellular staining using IntraPrep™ Permeabilization Reagent (Beckman Coulter, Fullerton, CA, USA) according to the manufacturer’s instructions. For the evaluation of surface glycosylation, cells were incubated with biotinylated lectins (Table S2 in Supplementary Material; Vector labs, Burlingame, CA, USA; EY labs, San Mateo, CA, USA) and HA-tagged, engineered chicken single chain, scFv-A4. The scFv-A4 has been demonstrated to be highly specific for the Gal-α-(1,3)-Gal terminal motif (24). The cells were washed with FACS buffer and labeled with fluorochrome-conjugated streptavidin (SA). For scFv-A4 labeling, an additional step of 15 min incubation with a biotinylated anti-HA antibody (Miltenyi Biotec, Bergisch Gladbach, Germany) was required, prior to the SA incubation. The resulting labeled cell suspensions were then either suspended in FACS buffer for flow cytometric analysis or in SORT buffer [DPBS (Gibco™), 1% FBS (Sigma-Aldrich), 25 mM HEPES solution, and 2 mM Ethylenediaminetetraacetic acid (Sigma-Aldrich)] for FACS. Flow cytometric analysis was performed using a BD FACSCanto™ II flow cytometer (BD Biosciences, San Jose, CA, USA), which was calibrated according to the manufacturer’s recommendations. Fluorescence compensation was set using single-stained controls, and matching median compensation algorithms were applied. Data were analyzed using Diva v8.0.1 (BD Biosciences) or FlowJo^®^ software (TreeStar Inc., Olten, Switzerland).

Fluorescence-activated cell sorting was performed for the purification of discrete cell populations using a BD FACSAriaII^®^ and BD FACSDiva^®^ v6 Software (BD Biosciences). Cell suspensions from mouse spleen and lymph nodes were labeled with the relevant fluorochrome-conjugated molecules then were washed, filtered through a 40-µm Sefar petex mesh and re-suspended in SORT buffer at the appropriate concentration (between 10 × 10^6^ and 40 × 10^6^ cells/ml) for separation. The preparation of human PBMC samples followed the same protocol, with initial enrichment of CD4^+^ T-cells by magnetic cell sorting using anti-human CD4 microbeads, MS columns and an OctoMACS^®^ separator according to manufacturer’s instructions (Miltenyi Biotec) prior to labeling for separation by FACS. In order to ensure only viable cells were collected during the purification, the appropriate viability dye (Molecular Probes™, Carlsbad, CA, USA) was added to the cell suspension immediately before sorting. At the end of the purification process, the purity of the isolated cells was determined by FCM analysis of an aliquot of each population. The purity of FACS-isolated cell populations used for the experiments described in the thesis was consistently between 92 and 98%.

### T-Cell Stimulation Cultures and Cocultures

Mouse cell cultures were carried out in complete culture medium. FACS-purified naive (CD4^+^CD62L^high^) mouse Tconv were cultured at 0.5 × 10^6^ cell/ml for 5 days at 37°C, 5% CO_2_ in 96-well round-bottom plates with and without plate-bound anti–mouse CD3ε (1 µg/ml) and anti-mouse CD28 (5 µg/ml) then were stained with combinations of biotinylated lectins and fluorochrome-coupled mAbs followed by fluorochrome-coupled SA and analyzed by multi-color FCM. For suppression assays, FACS-purified mouse Tconv were labeled with CellTrace^®^ Violet (Molecular Probes^®^) then cocultured for 1–4 days in 96-well round-bottom plates (Sarstedt, Nümbrecht, Germany) in complete culture medium with varying numbers of FACS-purified Treg in the presence or absence of FACS-purified CD4^−^CD8^−^ antigen-presenting cell (APCs) (4:1, APC:Tconv ratio) or CD11c^+^ dendritic cells (DCs) (1:3, DC:T-cell ratio) and of 0.1 µg/ml anti-mouse CD3ε (eBioscience). For APC-free activation, Mouse T-Activator CD3/CD28 Dynabeads^®^ (Gibco™) were added at 1:2 ratio to Treg/Tconv cocultures. FACS-purified human CD4^+^ Tconv were labeled with CellTrace^®^ Violet then cocultured for 4 days in 96-well round-bottom plates with FACS-purified (CD4^+^CD25^+^CD127^low^) Treg in RPMI 1640 Medium (Gibco™) supplemented with 10% FBS, 1% penicillin–streptomycin and 1% l-glutamine with or without Human T-Activator CD3/CD28 Dynabeads^®^ (Gibco™; 1:5 bead:Tconv ratio) prior to analysis by multi-color FCM.

### PNGase F Treatment of Treg

Fluorescence-activated cell sorting-purified mouse Treg were re-suspended in pre-warmed DPBS supplemented with 1 mM MgCl_2_ (Sigma-Aldrich), 1 mM CaCl_2_ (Sigma-Aldrich), and 1% BSA (Sigma-Aldrich) then plated at 0.2 × 10^6^ cells/well in 96-well flat-bottom plates (Sarstedt) to which 400 U/well of PNGase F (P0705L; New England Biolabs, Ipswich, MA, USA) were added to a final volume of 200 μl/well. After 1 h incubation at 37°C, 5% CO_2_, the cells were washed and re-suspended in complete culture medium. For the no enzyme controls, Treg were incubated under the same conditions in the absence of PNGase F.

### Analysis of Immune Cell Conjugates

Fluorescence-activated cell sorting-purified CellTrace™ CFSE-labeled Treg, CellTrace™ Violet-labeled (CD11c^+^) DC and CellTrace™ Far Red DDAO-SE-labeled (Molecular Probes™) CD4^+^ Tconv were admixed in 96-well round-bottom plates. The plates were briefly centrifuged at 450 RCF, incubated for 8 h at 37°C, 5% CO_2_ then washed with PBS and fixed for 5 min in 2% paraformaldehyde. Proportions of various multi-cell aggregate combinations were calculated based on FCM analysis of scatter and fluorescence characteristics (Figure S1 in Supplementary Material). For analysis of multi-cell conjugates by imaging FCM, the re-suspended cell pellets were additionally stained with Alexa Fluor^®^ 568 phalloidin (Molecular Probes™) following fixation/permeabilization then were analyzed using a FlowSight^®^ Imaging Flow Cytometer and IDEAS^®^ Software (Merck Millipore, Molsheim, France).

### Statistical Analysis

Individual experiments involved rare cell populations sorted to a high level of purity or required a large number of separate lectin stains per sample. As this frequently limited the number of replicates for each condition to between 3 and 6, precluding consistent testing of Gaussian distribution, statistical analyses were performed by permutation testing (25–27). Permutation testing using re-sampling methods was used to generate null-distributions of test-statistics for inference in accordance with published approaches (25, 26). This involved permuting the group labels for a given experimental factor and calculating the test statistic under each permutation. In all cases, the difference in means was used as the test statistic of interest and the observed difference was compared to the null-distribution of differences under permutation. If an experiment involved two factors in a factorial design, then re-sampling for inference on one factor was stratified within levels of the other factor and *vice versa*. If an experiment involved paired replicates, then the re-sampling was performed adhering to this paired design. The family-wise error rate within each experiment was controlled by applying the Holm correction to *p*-values pertaining to a single experimental factor (controlling for all pair-wise comparisons) (27). All unique permutations were used to generate each null-distribution, but if there were more than 10,000 unique permutations, a random sample of 10,000 was taken. These analyses were carried out using R (28). For experiments involving *n* = 3 or 4 replicates per experimental condition, results with *p* ≤ 0.1 were interpreted as being significant if the difference observed was also present in one or more similar experiments.

## Results

### Complex Tri/Tetra-Antennary *N*-Glycan Expression is Higher in Treg than in Tconv and is Increased with Activation

Glycosylation of mammalian cell surfaces is known to be dynamic and to undergo alterations at various developmental and differentiation stages, during physiological events such as cellular activation, migration, and apoptosis and also in the settings of cancer and inflammation (14, 17, 19, 29–33). The influence of surface glycosylation on T-cell development and function is well documented but there is a relative lack of existing knowledge regarding its specific relevance to Treg. For this reason, we initially set out to identify surface glycosylation characteristics which could potentially distinguish Treg from Tconv and to determine glycosylation changes that occur on the Treg surface following activation.

**Figure 1:**
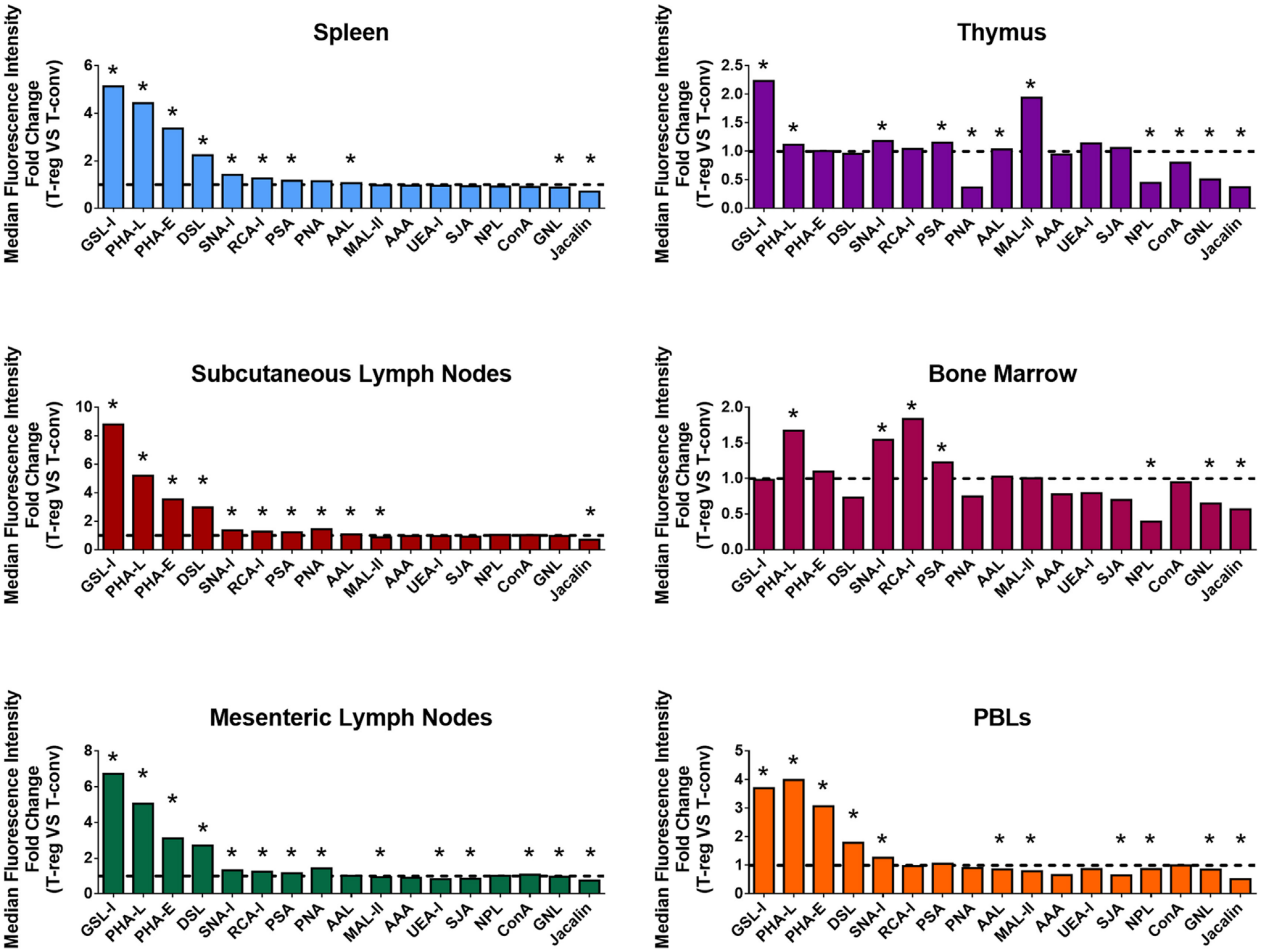
Differential surface lectin-binding intensities of Treg and CD4^+^ Tconv from various immunological niches in healthy mice. Surface glycosylation of freshly isolated CD4^+^ T-cells from C57BL/6 FoxP3.EGFP mouse spleen, subcutaneous lymph nodes, mesenteric lymph nodes, thymus, bone marrow, and peripheral blood leukocytes (PBLs) was evaluated by lectin profiling using flow cytometry as described in the Section “Materials and Methods.” Results are presented as the fold change in median fluorescence intensity (MFI) of lectin binding between Treg and Tconv (*n* = 4–8 individual animals). Line at *y* = 1 represents the value at which the lectin-binding intensities are the same for Treg and CD4^+^ Tconv. Statistical analysis was performed by permutation test with a paired design (*p value ≤ 0.1).

In order to broadly compare surface glycosylation characteristics of Treg and CD4^+^ Tconv, cell suspensions from the major lymphoid organs and PBL of C57BL/6 FoxP3.EGFP mice were analyzed by multicolor FCM using a panel of 17 biotinylated lectins (Figure 1; with full dataset provided Table S1 in Supplementary Material). At each of these sites, GFP^+^ Treg displayed distinct lectin-binding characteristics when compared to Tconv (Figure 1). Of note, the hierarchies of differential lectin-binding intensities for Treg compared to Tconv were similar for spleen, subcutaneous lymph nodes, mesenteric lymph nodes, and PBL, although the magnitudes of these differences were lower for PBL (Figure 1). Based on the recognized carbohydrate affinities of the lectins employed (Table S1 in Supplementary Material), these results indicated that the surface glycosylation profile of Treg at these mouse secondary lymphoid sites includes several specific features when compared to Tconv: higher *Datura stramonium* lectin (DSL), *Phaseolus vulgaris* erythroagglutinin (PHA-E), PHA-L, and *Ricinus communis* lectin I (RCA-I) binding, suggestive of broadly higher expression of *N*-acetylglucosamine (GlcNAc)-containing carbohydrate structures, with or without terminal β-Gal; higher *Griffonia simplicifolia* lectin I (GSL-I) binding and lower Jacalin binding, suggestive of higher expression of terminal galactose (Gal) and *N*-acetylgalactosamine (GalNAc) residues attached in α linkage and lower expression of sialylation-tolerant Gal residues, respectively; higher *Sambucus nigra* lectin I (SNA-I) binding and lower *Maackia amurensis* lectin II (MAL-II) binding, suggestive of higher expression of α-(2,6) linked sialic acid and lower expression of α-(2,3)-linked sialic acid, respectively; higher *Pisum sativum* agglutinin (PSA) binding suggestive of higher expression of fucose (Fuc)-dependent terminal mannose (Man) containing glycan structures and higher *Aleuria aurantia* lectin (AAL) binding, suggestive of higher expression of α-(1,6)-linked Fuc residues. In contrast, the differential lectin-binding characteristics of Treg and Tconv in primary lymphoid sites (thymus and bone marrow) exhibited different features (Figure 1; Table S1 in Supplementary Material), likely reflecting the influence of glycosylation events linked to lymphocyte development and hematopoiesis at these sites.

**Figure 2:**
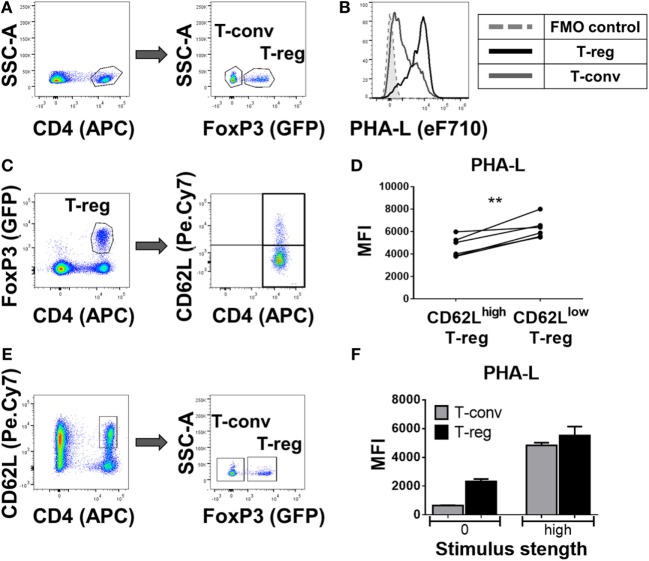
Complex tri/tetra-antennary *N*-glycan expression is higher in Treg than Tconv and is upregulated upon activation stimuli. **(A)** Flow cytometric identification of CD4^+^FoxP3^+^ Treg (GFP^+^) and CD4^+^FoxP3^−^ Tconv (GFP^−^) from C57BL/6 FoxP3.EGFP mouse spleen. **(B)** Comparison of *Phaseolus vulgaris* leucoagglutinin (PHA-L) binding to freshly isolated Treg and Tconv. **(C)** Gating strategy for the flow cytometric analysis of the CD62L^high^ and CD62L^low^ GFP^+^ Treg subsets. **(D)** Graph representing the Median Fluorescence Intensity (MFI) of PHA-L binding to CD62L^high^ and CD62L^low^ GFP^+^ Treg subsets (*n* = 6 individual animals). **(E)** Gating strategy of FACS purification of naïve CD4^+^CD62L^high^ T-cells (left) and subsequent analysis of GFP^−^ Tconv and GFP^+^ Treg. **(F)** MFIs of PHA-L binding to Tconv and Treg following 5-day culture of purified CD4^+^CD62L^high^ T-cells with no stimulus (0) or with anti-CD3/CD28 mAb stimulus (high). Results shown are representative of three similar experiments (*n* = 3 technical replicates per condition). Statistical analysis was performed by permutation test with a paired design (**p value < 0.05).

In a more detailed analysis of spleen, the surface level of tri/tetra-antennary complex *N*-glycan expression (based on PHA-L binding intensity) was strikingly higher on Treg compared to CD4^+^ Tconv (Figures 2A,B) and was higher on previously activated (CD62L^low^) compared to naïve (CD62L^hi^) Treg (Figures 2C,D). When CD62L^hi^ CD4^+^ T-cells were FACS purified and placed in culture with no stimulus for 5 days, differential binding of PHA-L to Treg and Tconv was maintained. However, following anti-CD3/anti-CD28 stimulation, increased PHA-L binding was observed in both Treg and Tconv (Figures 2E,F). Thus, mouse CD4^+^ T-cell surface glycosylation varies among the lymphoid organs and PBL with Treg surface glycosylation being distinct from that of Tconv at each site. The observed differences in PHA-L binding intensity suggest that tri/tetra-antennary complex *N*-glycan expression is consistently higher in freshly isolated Treg but increases to similar levels on Tconv following activation through the TCR and CD28.

### Complex Tri/Tetra-Antennary *N*-Glycan Surface Expression Correlates with Expression Levels of Treg Surface Markers

**Figure 3:**
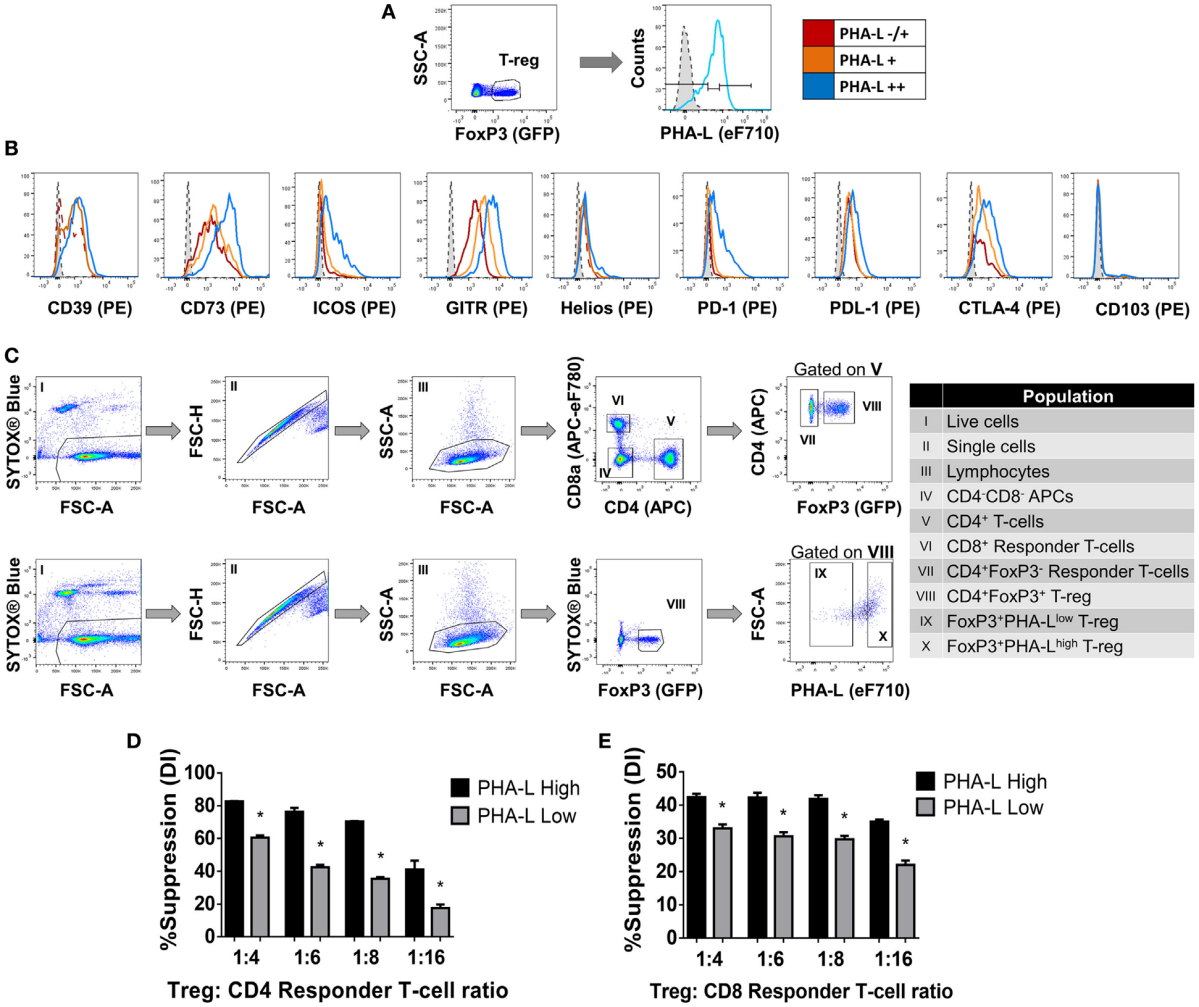
Complex tri/tetra-antennary *N*-glycan surface expression correlates with expression levels of Treg markers and suppressive potency. **(A,B)** The expression of CD39, CD73, ICOS, GITR, Helios, PD-1, PDL-1, CTLA-4, and CD103 was evaluated in Treg subpopulations within freshly isolated spleen and lymph node cells from C57BL/6 FoxP3.EGFP mice defined according to *Phaseolus vulgaris* leucoagglutinin (PHA-L) binding intensities [low (−/+), mid (+) or high (++)]. Dashed tinted histograms represent the median fluorescence intensities for the eF710 and PE fluorescence minus one (FMO) controls for lectin and antibody labeling, respectively (*n* = 3 individual animals). **(C)** Gating strategy for FACS purification of cell populations used in the functional assay **(D,E)**. **(D)** CD4^+^ and **(E)** CD8^+^ responder T-cells were labeled with CellTrace™ Violet and cocultured in the presence of CD4^−^CD8^−^ antigen-presenting cells and purified PHA-L^high^ or PHA-L^low^ Treg at Treg: responder T-cell ratios of 0:1, 1:4, 1:6, 1:8, and 1:16 for 4 days with anti-CD3 stimulation. Suppressive function was quantified for PHA-L^high^ and PHA-L^low^ Treg based on responder T-cell division index (DI) and presented as the calculated percent suppression [%Suppression (DI) = 100 − [(DI of the 1:*X*)/(DI of 0:1)] × 100 in which 1:*X* represent cocultures with Treg and 0:1 refers to cocultures with responder T-cell alone]. Data represent mean ± SD (*n* = 3 technical replicates). Results for proliferative assays were confirmed in a repeat experiment. Statistical analysis was performed by permutation test with an unpaired design (*p value ≤ 0.1).

Among the lectins evaluated, GSL-I and PHA-L demonstrated the most striking differential staining patterns for mouse Treg compared to Tconv (Figure 1). Furthermore, higher Treg surface binding of GSL-I and PHA-L correlated with higher Treg expression of putative suppressive mediators. However, the biological relevance of the GSL-I ligand cannot be translated from mouse to human since humans are not equipped with the enzyme required for the biosynthesis of this glycan structure (34). In addition, the degree of branching of *N*-glycans, and thus the expression of PHA-L ligands (tri-/tetra-antennary *N*-glycans), have been described to play important roles in modulating glycoprotein activity, localization, mobility, and clustering. The observed remodeling of the Treg surface glycome according to anatomical location and activation state suggested that Treg glycosylation may play an important role in modulating the suppressive function of these cells. To address this, the relationships between expression of functionally relevant Treg surface proteins and surface density of tri/tetra-antennary complex *N*-glycans (based on binding intensity of PHA-L) were investigated. Treg were subdivided into three subpopulations based on lectin staining intensity [PHA-L negative (^−^), low/mid (^−/+^) and high (^++^)] (Figure 3A) and the expression levels of a panel of Treg markers were compared among these subpopulations. As shown in Figure 3B, PHA-L binding level correlated with higher expression of CD39, CD73, ICOS, GITR, PD-1, PD-L1 and CTLA-4—each of which is reported to play an important role in Treg development and suppressive functions (3–5, 35).

**Figure 4:**
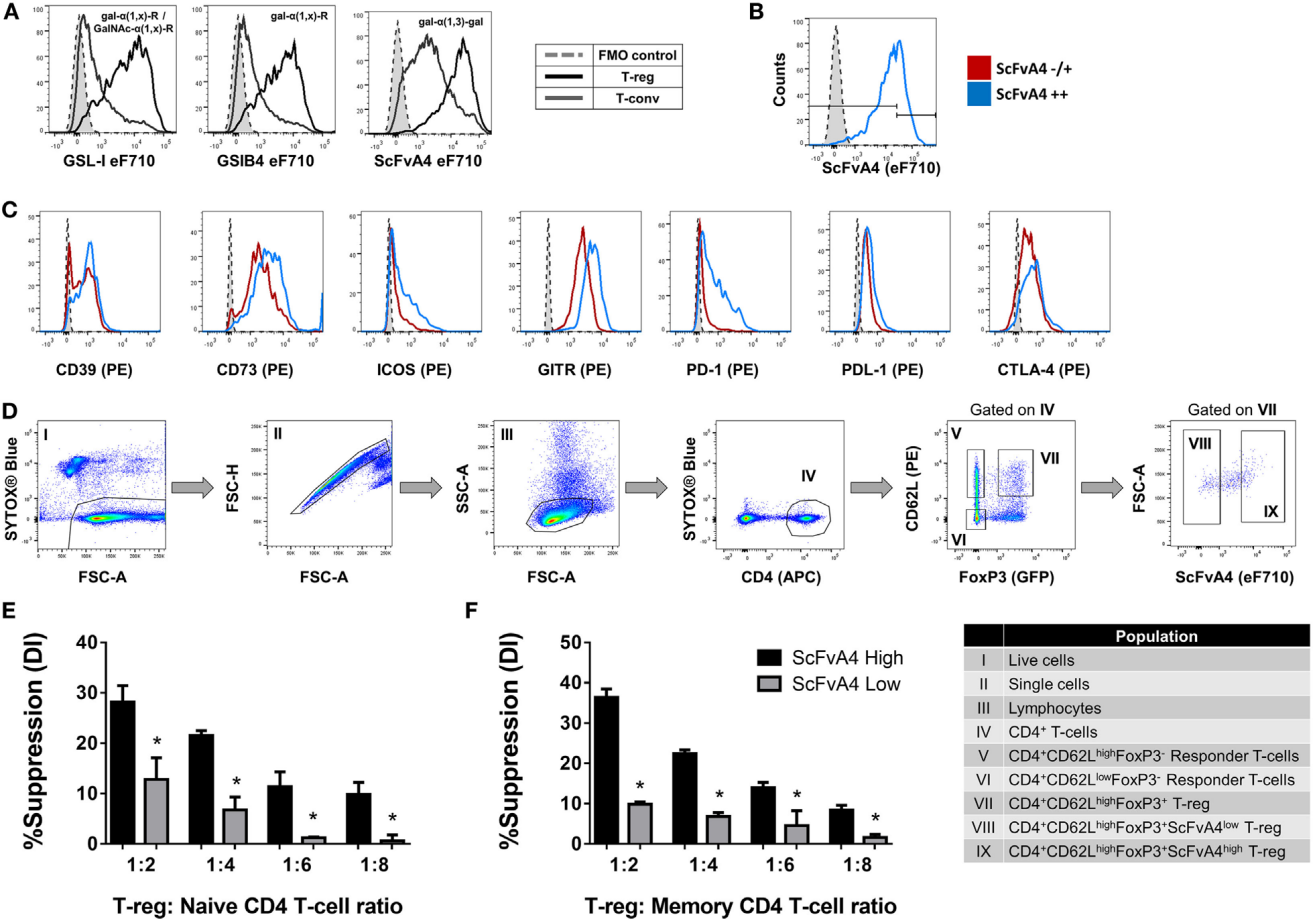
Terminal alpha-galactose surface expression correlates with expression levels of Treg markers and suppressive potency. **(A)** Flow cytometric analysis of the surface binding intensities of α-Gal binding proteins [*Griffonia simplicifolia* lectin I (GSL-I), GSLB4, and anti-Gal-α-(1,3)-Gal scFv-A4] to Treg and Tconv. **(B,C)** Flow cytometric analysis of the expression of CD39, CD73, ICOS, GITR, PD-1, PDL-1, and CTLA-4 on Treg subpopulations defined according to low and high binding intensities of anti-Gal-α-(1,3)-Gal scFv-A4 [scFv-A4^−/+^ or scFv-A4^++^]. Dashed histograms represent the median fluorescence intensity of the fluorescence minus one (FMO) controls (*n* = 3 individual animals). **(D)** Gating strategy for purification of scFv-A4^high^ and scFv-A4^low^ Treg subpopulations from C57BL/6 FoxP3.EGFP mouse spleen and lymph nodes. Individual sorted populations are identified by Roman numerals and described in the text box. **(E,F)** Suppressive function was quantified based on responder T-cell division index (DI) and presented as the calculated percent suppression [%Suppression (DI)] for scFv-A4^high^ and scFv-A4^ow^ Treg of **(E)** naive and **(F)** memory CD4^+^ responder T-cell proliferation. Data represent mean ± SD of (*n* = 3 technical replicates). Results for proliferative assays were confirmed in a repeat experiment. Statistical analysis was performed by permutation test with an unpaired design (*p value ≤ 0.1).

Next, to determine whether tri/tetra-antennary complex *N*-glycan expression also correlates with Treg function, FACS-purified PHA-L^low^ and PHA-L^high^ Treg were compared in an *in vitro* suppression assay (Figure 3C). Purified PHA-L^high^ Treg displayed increased potency in suppressing proliferation of CD4^+^ and CD8^+^ Tconv when compared to purified PHA-L^low^ Treg (Figures 3D,E). Importantly, the observed correlation between glycan surface expression and Treg expression of functionally relevant molecules was not confined to tri/tetra-antennary complex *N*-glycans. For example, higher binding to Treg compared to Tconv was also observed for the lectins GSL-I and GSLB4 (Figure 4A). These lectin-binding characteristics were shown to reflect surface levels of the terminal glycan motif composed of a Gal residue in α-(1,3)-linkage to another Gal residue (Gal-α-(1,3)-Gal) using a single-chain fragment with specificity for this motif (scFv-A4) (24). As for PHA-L, Treg with higher scFv-A4 binding exhibited higher surface expression of CD73, ICOS, GITR, and PD-1 (Figures 4B,C). Moreover, purified scFv-A4^high^ Treg (Figure 4D) had greater capacity to suppress the proliferation of naïve and memory CD4^+^ Tconv than their scFv-A4^low^ counterparts (Figures 4E,F). Thus, in mouse, higher tri/tetra-antennary complex *N*-glycan and Gal-α-(1,3)-Gal terminal motif expression correlates with increased Treg suppressive potency.

### Manipulation of Treg Surface *N*-Glycosylation Affects Suppressive Potency

**Figure 5:**
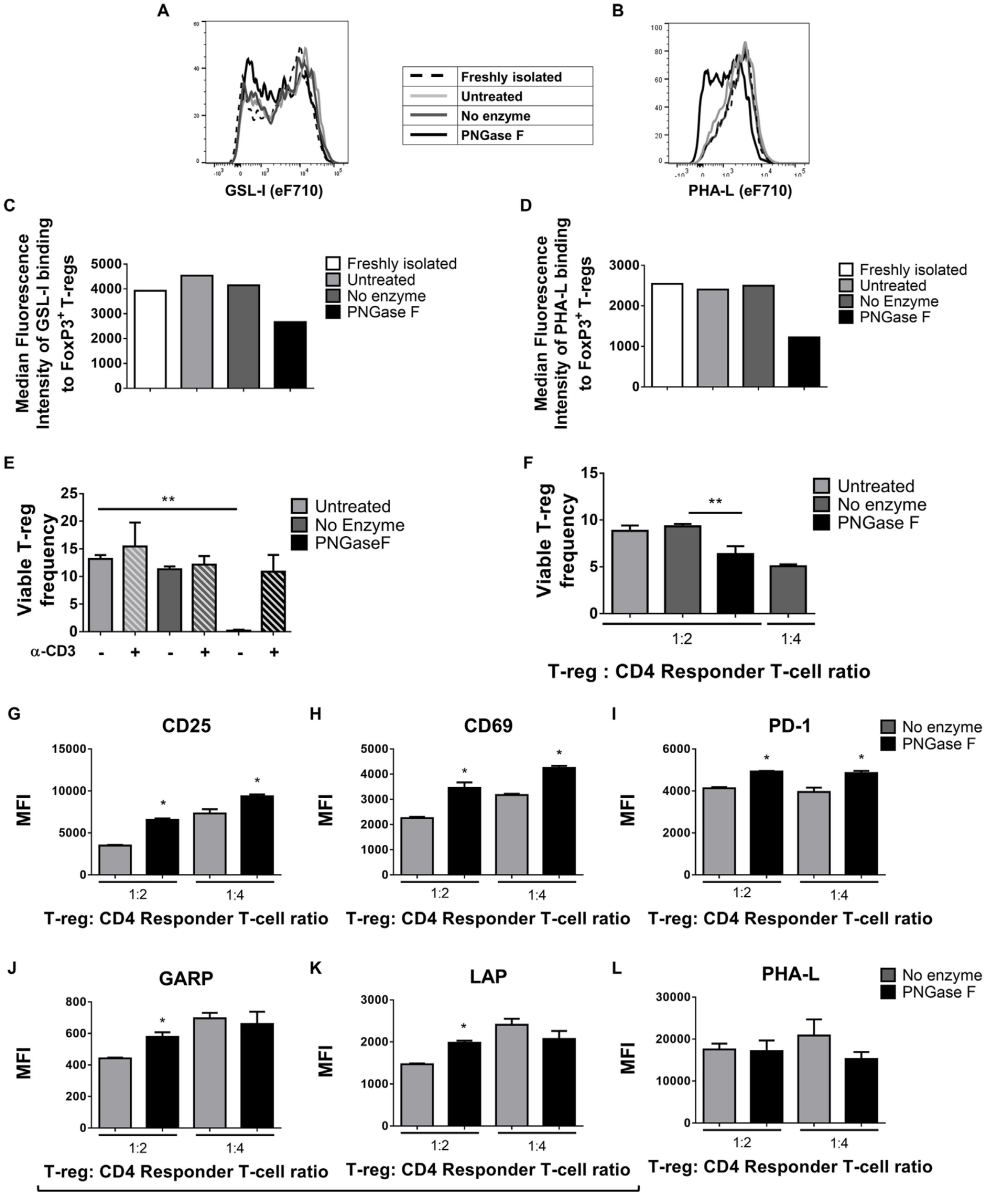

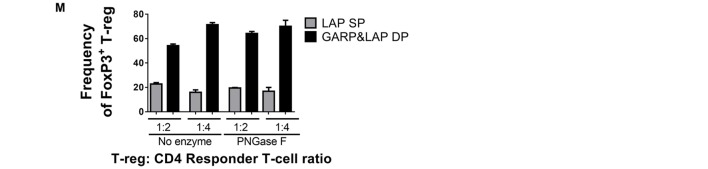
Surface *Griffonia simplicifolia* lectin I (GSL-I) and *Phaseolus vulgaris* leucoagglutinin (PHA-L) binding, viability, and responsiveness to stimulus of PNGase F-treated Treg. **(A–D)** Flow cytometry (FCM) histogram overlays and graphs of median fluorescence intensity (MFI) of GSL-I **(A,C)** and PHA-L **(B,D)** binding to freshly isolated Treg, Treg incubated in culture medium alone (Untreated), Treg incubated in enzyme buffer alone (no enzyme) and Treg incubated in enzyme buffer containing PNGase F. For the latter three, analyses were performed immediately after incubations. **(E)** Frequencies of viable Treg after 24 h coculture with antigen-presenting cells (APCs) with and without anti-CD3 activation assessed by FCM using SYTOX^®^ AADvanced dead cell stain. **(F–M)** Treg were cocultured with CD4^+^ Tconv at 0:1, 1:2, and 1:4 Treg: responder T-cell ratios in the presence of APCs and anti-CD3 activation for 24 h, after which **(F)** the frequency of viable Treg and **(G–M)** Treg expression of several surface proteins were evaluated. Graphs show surface expression levels (MFI) of **(G)** CD25, **(H)** CD69, **(I)** PD-1, **(J)** GARP, **(K)** LAP, and **(L)** complex tri/tetra-antennary *N*-glycans (PHA). **(M)** Frequency of LAP single positive (LAP SP) and GARP and LAP double positive (GARP&LAP DP) Treg after 24 h coculture. **(A–D)** Data are representative of one of five separate experiments. **(E–M)** Data represent mean ± SD (*n* = 3 technical replicates). Statistical analysis was performed by permutation test with an unpaired design (*p value ≤ 0.1; **p value < 0.05).

**Figure 6:**
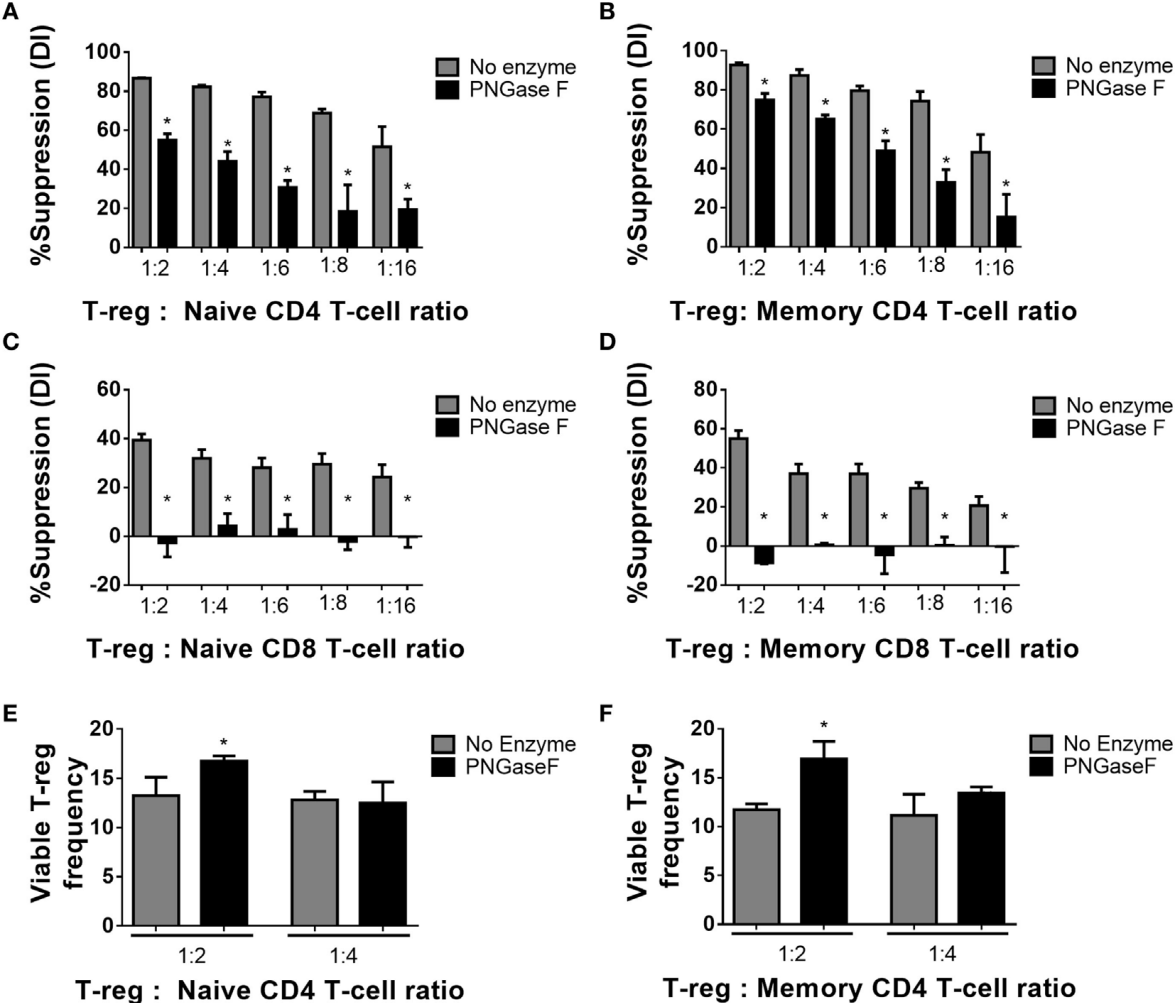
Manipulation of murine Treg surface *N*-glycosylation impairs suppressive potency. **(A)** Naïve and **(B)** memory CD4^+^ Tconv and **(C)** naïve and **(D)** memory CD8^+^ Tconv were cocultured in the presence of CD4^−^CD8^−^ antigen presenting cells and no enzyme or PNGase F-treated Treg populations at Treg: responder T-cell ratios of 0:1, 1:2, 1:4, 1:6, 1:8, and 1:16 for 4 days with anti-CD3 stimulation. Suppressive function was quantified based on responder T-cell division index (DI) after 4 days and presented as the calculated percent suppression [%Suppression (DI)]. **(E,F)** Day 4 frequencies of viable Treg were expressed as percentages of the total viable cells (Viable Treg frequency) in the cocultures with **(E)** naïve and **(F)** memory CD4^+^ responder T-cells. Data represent mean ± SD (*n* = 3 technical replicates). Results were confirmed in three independent experiments. Statistical analysis was performed by permutation test with an unpaired design comparing the coculture with the different Treg populations to the no Treg condition (*p value ≤ 0.1).

**Figure 7:**
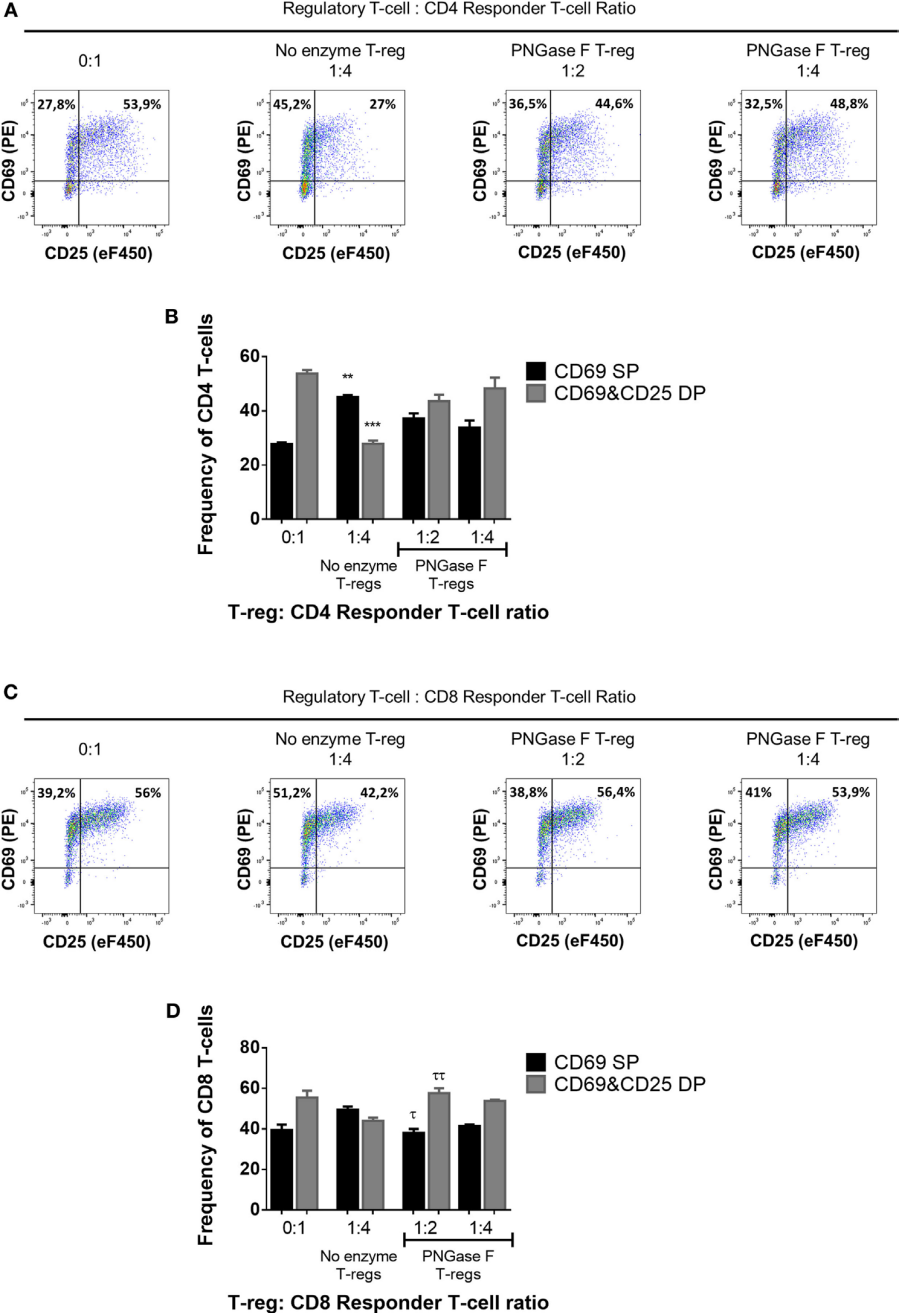
Manipulation of surface *N*-glycosylation impairs the early suppressive potency of murine Treg. **(A–D)** Fluorescence-activated cell sorting-purified CD4^+^ **(A,B)** and CD8^+^ **(C,D)** Tconv were cocultured in the presence of CD4^−^CD8^−^ antigen-presenting cells without Treg (0:1) and with no enzyme or PNGase F-treated Treg at Treg: responder T-cell ratios of 1:2 and 1:4 for 24 h with anti-CD3 stimulation. Suppressive function was evaluated based on inhibition of **(A,B)** CD4^+^ and **(C,D)** CD8^+^ responder T-cell upregulation of CD25 and CD69 after 24 h coculture and is presented as the frequencies of CD69 single positive (CD69 SP) and CD69 and CD25 double positive (CD69&CD25 DP) cells for cocultures containing no enzyme and PNGase F-treated Treg. **(A,C)** Representative flow cytometric dot plots of the expression of the activation markers CD25 and CD69 on Tconv populations. The frequencies of CD69 SP (top left quadrant) and CD69 and CD25 double positive (top right quadrant) Tconv are shown for four coculture conditions: no Treg (0:1); no enzyme Treg at 1:4 Treg: responder T-cell ratio; PNGase F-treated Treg at 1:2 Treg: responder T-cell ratio and PNGase F-treated Treg at 1:4 Treg: responder T-cell ratio. **(B,D)** Graphs showing the relative distribution and frequency of cells with differential CD69 and CD25 expression in the different culture conditions. Data represent mean ± SD (*n* = 3 technical replicates). Results were confirmed in three independent experiments. Statistical analysis was performed by permutation test with an unpaired design comparing the coculture with the different Treg populations to the no Treg condition (*p value ≤ 0.1; **p value < 0.05; ***p value < 0.01) and between the PNGase F and the no enzyme Treg cocultures (^τ^*p* value ≤ 0.1; ^ττ^*p* value < 0.05).

Thus far, the observations suggested that the level of surface glycan expression (as determined by PHA-L and scFv-A4 binding intensities) identified Treg with differential suppressive potency. Next, we asked whether *N*-glycans at the Treg surface play an active role in Treg suppression of Tconv activation. To address this question, suppression assays were performed using FACS-purified splenic Treg in which surface *N-*glycosylation was enzymatically manipulated. A short incubation with PNGase F resulted in decreased surface binding of GSL-I and PHA-L [suggestive of lower expression of terminal α-Gal/GalNAc residues and tri/tetra-antennary complex *N*-glycans, respectively] without compromising Treg viability and responsiveness to activation stimuli (Figure 5). However, when compared to control (no enzyme) Treg, PNGase F-treated Treg exhibited lower anti-proliferative potency for naïve and memory CD4^+^ (Figures 6A,B) and CD8^+^ Tconv (Figures 6C,D). Importantly, evaluation of Treg after the 4-day suppression assay indicated that the PNGase F-treated Treg were present at comparable or greater frequency to untreated (“no enzyme”) Treg (Figures 6E,F). Furthermore, when compared to no enzyme Treg, PNGase F-treated Treg had impaired ability to inhibit Tconv upregulation of CD69 and CD25 during the first 24 h of activation culture—particularly the transition from CD69^+^CD25^−^ to CD69^+^CD25^+^ (Figure 7). It was concluded that partial loss of Treg surface *N*-glycan expression substantially impairs splenic Treg suppression of early activation events and subsequent proliferation of naïve and memory CD4^+^ and CD8^+^ Tconv. In keeping with the disruption of early events, surface expression of PHA-L ligands was no different between PNGase F-treated and control Treg by 24 h (Figure 5L).

### PNGase F-Treated Treg Retain the Ability to Interact with DCs and Tconv

The activation of T-cells by APCs is known to require a stable interaction of molecules at the intercellular interface—termed the immunological synapse—where signaling, secretion, and endocytosis events are focused (36). Recent real-time imaging studies have shown that Treg engage in interactions with effector T-cell populations *in vivo*, both with and without DC involvement (37, 38). The glycosylation levels of surface proteins have been proposed to influence the formation of stable immunological synapses by controlling the alignment of opposing cell surfaces and regulating the geometry of the interactions between proteins as well as the stability of the individual molecules at the synapse (39–41). As the *in vitro* anti-proliferative effect of purified mouse Treg was not reproduced by transfer of conditioned medium from Treg/Tconv cocultures to fresh Tconv stimulation cultures (data not shown), we concluded that the suppressive mechanisms at play in these cultures is contact-dependent.

**Figure 8:**
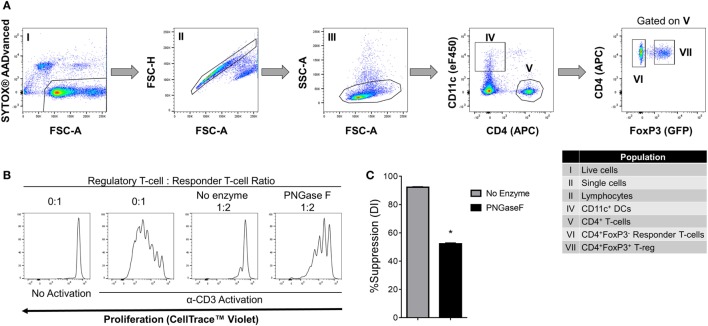
Suppression of CD4^+^ responder T-cell proliferation by PNGase F-treated Treg in the presence of dendritic cells (DC). **(A)** Gating strategy for purification of CD11c^+^ DCs, CD4^+^ Tconv, and Treg from C57BL/6 FoxP3.EGFP mouse spleen and lymph nodes. Individual sorted populations are identified by Roman numerals and described in the text box. **(B,C)** Fluorescence-activated cell sorting (FACS)-purified CD4^+^ Tconv were cocultured in the presence of FACS-purified CD11c^+^ DCs without Treg (0:1) and with no enzyme or PNGase F-treated Treg at Treg: responder T-cell ratio 1:2 for 4 days with anti-CD3 stimulation. **(B)** Proliferation of CellTrace™ Violet-labeled CD4^+^ Tconv analyzed by flow cytometry at the end of the culture period. **(C)** Graph of the suppressive potency of no enzyme and PNGase F-treated Treg on CD4^+^ Tconv. Suppressive function was quantified based on Tconv division index (DI) and presented as the calculated percent suppression [%Suppression (DI)]. Data represent mean ± SD (*n* = 3 technical replicates). The experiments shown in panels **(B,C)** are representative of three independent experiments with consistent results. Statistical analysis was performed by permutation test with an unpaired design (*p value ≤ 0.1).

**Figure 9:**
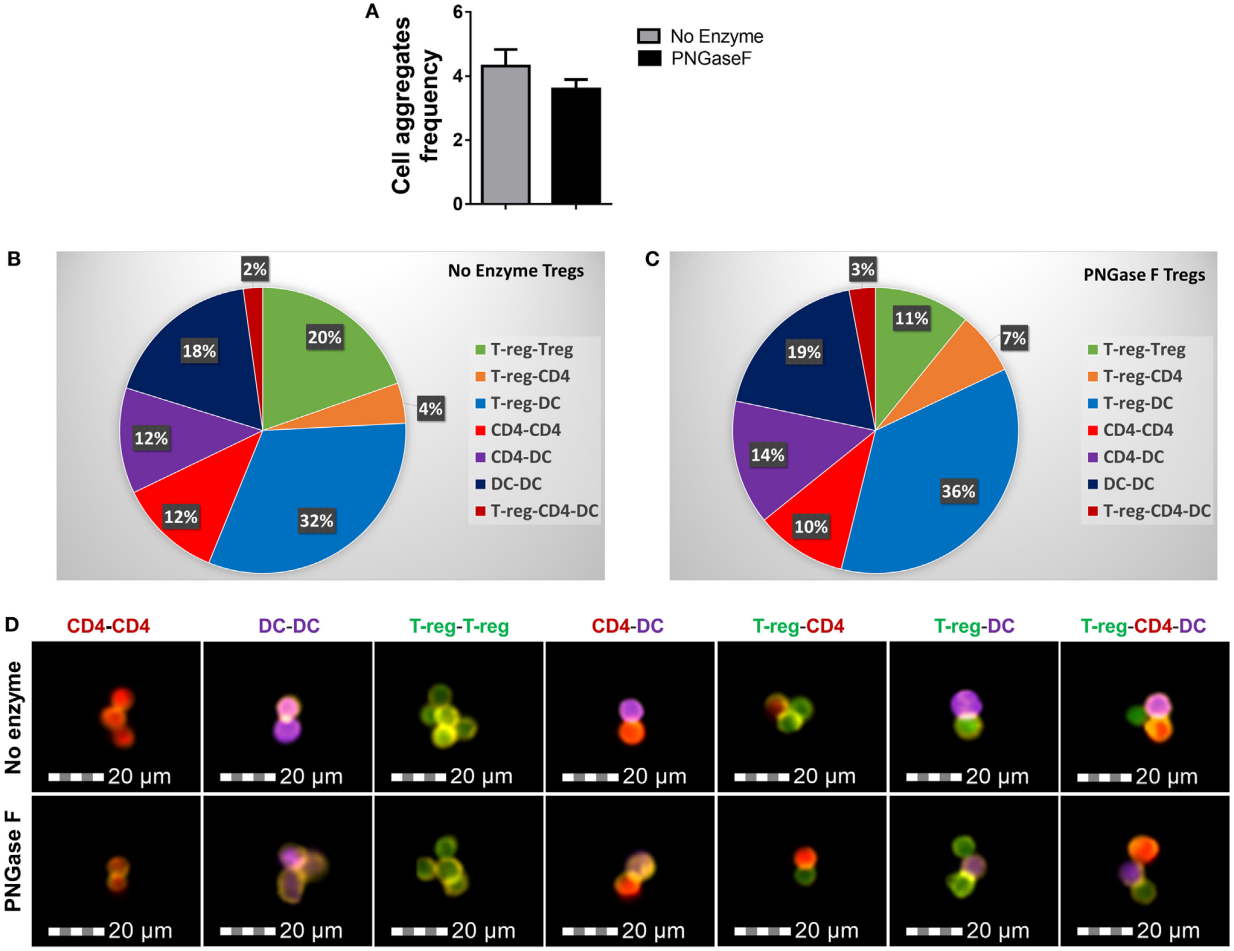
PNGase F treatment does not affect Treg ability to interact with other immune cells. **(A–D)** CD4^+^ responder T-cells were cocultured with CD11c^+^ dendritic cells (DCs) and purified Treg (no enzyme or PNGase F-treated) at Treg: responder T-cell ratios of 1:2 for 8 h with anti-CD3 stimulation. Prior to coculture, Treg were fluorescently labeled with CellTrace™ CFSE (CFSE), DCs with CellTrace™ Violet (CTV) and CD4^+^ Responder T-cells with CellTrace™ Far Red DDAO-SE (Far red). Immune cell interactions were quantified by flow cytometry. **(A)** Graph of the frequency of cell aggregates and **(B,C)** pie charts showing the proportionate frequencies of the different types of multi-cell aggregates present in the cocultures with **(B)** no enzyme and **(C)** PNGase F-treated Treg. **(D)** Imaging flow cytometric analysis of the immune cell interactions. Immunological synapse formation was identified by selective F-actin staining with Alexa Fluor^®^ 568 phalloidin (yellow stain). Representative examples of different types of immune cell aggregates present in the cocultures are presented for no enzyme and PNGase F-treated Treg cocultures. Data for **(A–C)** represent overall mean result from three identical experiments each with three technical replicates per condition (*n* = 9). Statistical analysis was performed by permutation test with an unpaired design and no significant differences were identified.

**Figure 10:**
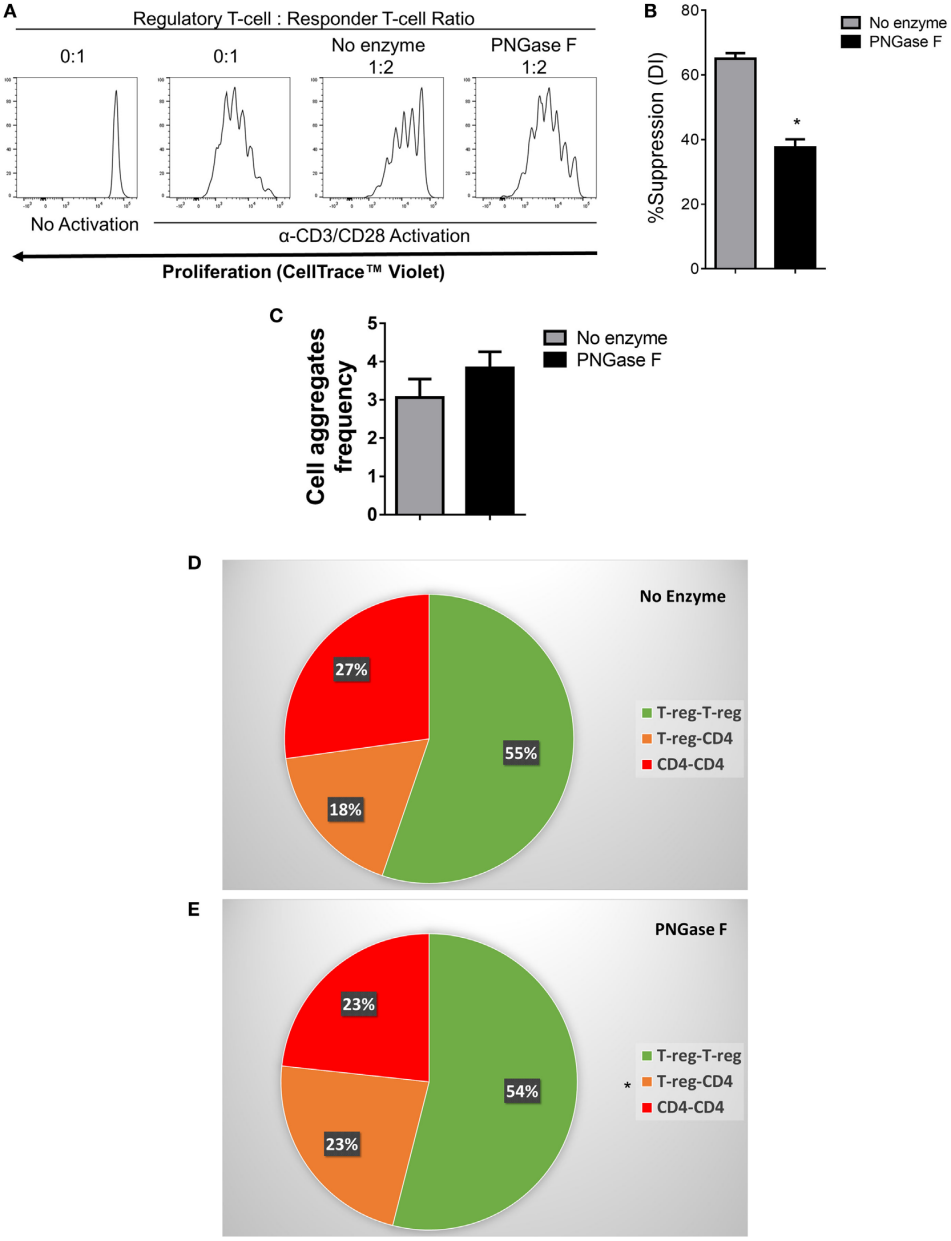
Flow cytometric analysis of the suppression of CD4^+^ responder T-cell proliferation and immune cell interactions established by PNGase F-treated Treg in the absence of antigen presenting cells. **(A–E)** Fluorescence-activated cell sorting (FACS)-purified mouse CD4^+^ Tconv were cultured alone (0:1) or cocultured in the presence of no enzyme or PNGase F-treated Treg at Treg: responder T-cell ratios of 0:1 and 1:2 for 4 days **(A,B)** or 8 h **(C–E)** with anti-CD3/CD28 Dynabeads^®^ stimulation. **(A)** Proliferation of CellTrace™ Violet-labeled CD4^+^ Tconv incubated under different conditions and analyzed by flow cytometry. **(B)** Graph of the suppressive potency of no enzyme and PNGase F-treated Treg on CD4^+^ T-cell responders. Suppressive function was quantified based on responder T-cell division index (DI) and presented as the calculated percent suppression [%Suppression (DI)]. For evaluation of immune cell interactions in 8 h cocultures, FACS-purified cells were fluorescently labeled as follows: Treg with CellTrace™ CFSE and CD4^+^ Tconv with CellTrace™ Far Red DDAO-SE. **(C–E)** Flow cytometric quantification of the interactions present in the cocultures at 8 h presented as **(C)** a graph of the overall frequency of multi-cell aggregates and **(D,E)** pie charts showing the calculated frequencies of the different types of multi-cell aggregates present in the cocultures with **(D)** no enzyme and **(E)** PNGase F-treated Treg. Data represent mean ± SD (*n* = 3 technical replicates). Statistical analysis was performed by permutation test with an unpaired design (*p value ≤ 0.1).

We, therefore, proceeded to investigate the effects of PNGase F treatment on Treg ability to interact with DC and CD4^+^ Tconv. Initially, it was confirmed that replacement of the mixed splenic APC population with purified splenic CD11c^+^ DC was associated with similar suppression assay characteristics (Figure 8). Next, flow cytometric evaluation of multi-cell aggregates from activated DC/Tconv/Treg cocultures after 8 h (Figure S1 in Supplementary Material) revealed that PNGase F-treated Treg exhibited similar ability to form conjugates with DC and CD4^+^ T-cells as the no enzyme control Treg (Figure 9). The integrity of the multi-cell aggregates was further established by imaging FCM which demonstrated phalloidin^+^ interfaces between cells of the various types, indicative of F-actin polymerization at the points of contact (Figure 9D). Finally, similar observations regarding loss of Treg suppressive potency with maintenance of the ability to interact with CD4^+^ Tconv following PNGase F modulation were also obtained in APC-free activation cultures (Figure 10). The combined evidence from these experiments indicated that the observed loss of Treg suppressive potency following PNGase F treatment was not explained by impaired ability to form stable conjugates with DCs or Tconv.

### Complex Tri/Tetra-Antennary *N*-Glycan Surface Expression Correlates with Human Treg Suppression of Naïve CD4^+^ T-Cells

**Figure 11:**
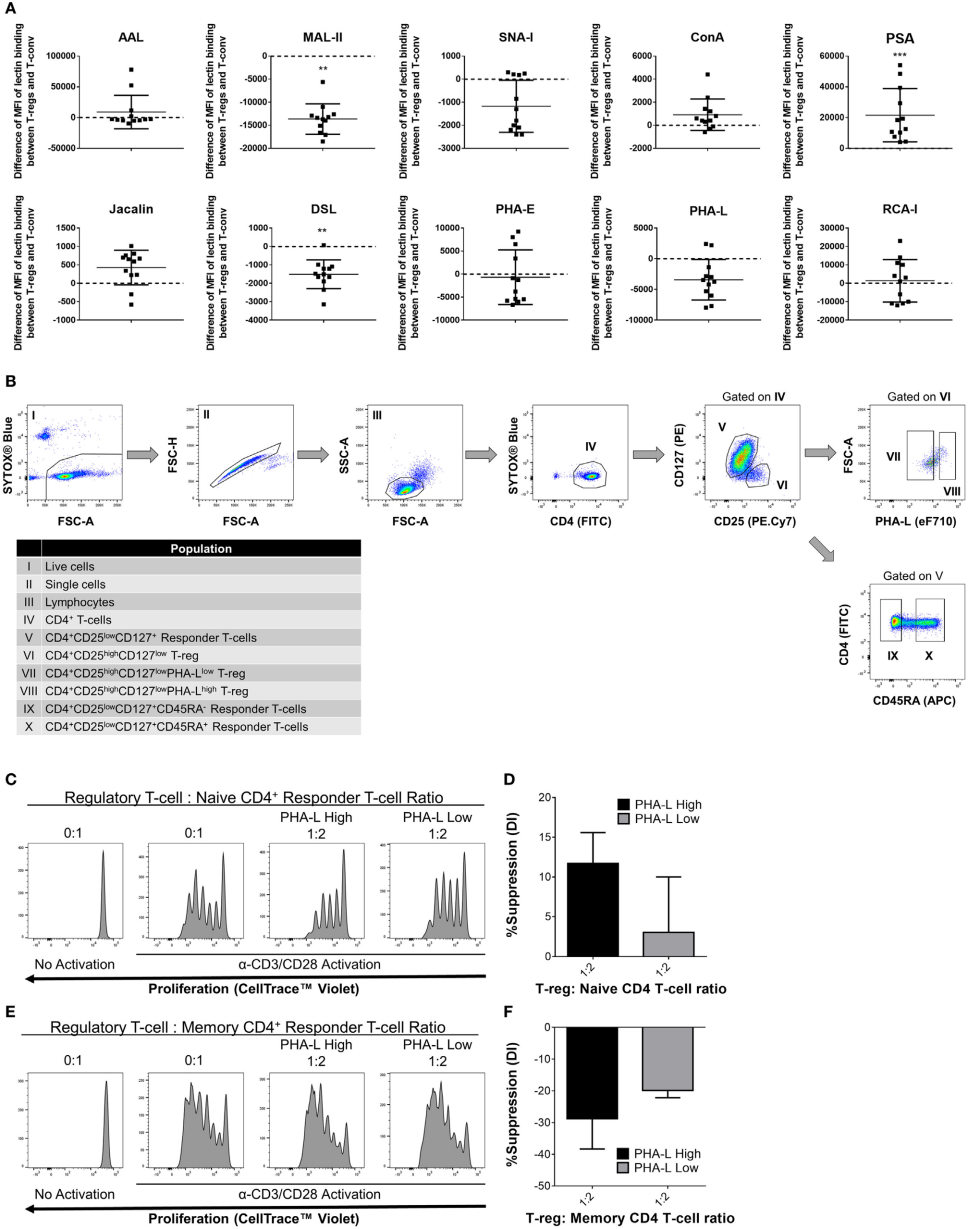
Complex tri/tetra-antennary *N*-glycan surface expression correlates with human Treg suppressive potency. **(A)** Surface glycosylation of human peripheral blood mononuclear cell (PBMC) was evaluated by lectin profiling using flow cytometry. Results are shown as the difference in Median Fluorescence Intensity (MFI) of lectin binding between CD4^+^CD25^+^CD127^low^ Treg and CD4^+^CD25^−/low^CD127^+^ Tconv and are representative of results from freshly isolated PBMC (*n* = 12 individual subjects). Statistical analysis was performed by permutation test with a paired design (*p value ≤ 0.1; **p value < 0.05; ***p value < 0.01). **(B)** Gating strategy for fluorescence-activated cell sorting purification of human PBMC-derived immune cell populations for functional assays. Individual sorted populations are identified by Roman numerals and described in the text box. **(C–F)** Naïve CD45RA^+^ **(C,D)** and memory CD45RA^−^ **(E,F)** CD4^+^ responder Tconv were cocultured in the presence of purified PHA-L^high^ and PHA-L^low^ Treg at 0:1 and 1:2 Treg: responder T-cell ratios for 4 days with anti-human CD3/CD28 Dynabeads^®^ stimulation. **(C,E)** Histograms showing representative examples of responder Tconv proliferation evaluated by flow cytometric analysis of CellTrace™ Violet dilution. **(D,E)** Graphs of the suppressive potency of PHA-L^high^ and PHA-L^low^ Treg on naïve and memory CD4^+^ T-cell responders. Suppressive function was quantified based on responder T-cell division index (DI) and presented as the calculated percent suppression [%Suppression (DI)]. Data represent mean ± SD (*n* = 3 technical replicates). Results were confirmed in a repeat experiment. Statistical analysis was performed by permutation test with an unpaired design and no significant differences were identified.

Based on the observations in mouse, it was hypothesized that human Treg surface glycosylation characteristics are also distinct from those of Tconv. Indeed, lectin profiling of T-cells within human PBMC preparations from 12 healthy adults revealed distinct surface glycosylation differences between CD4^+^ CD25^+^ CD127^low^ Treg and CD4^+^ Tconv (Figure 11A). Similar to mouse, human Treg showed higher binding by PSA and lower binding by MAL-II than their Tconv counterparts. However, in contrast to mouse, human Treg had lower binding of DSL. The remainder of the lectins either showed no differential binding or binding trends that were opposite to those observed for the same lectin on mouse PBLs.

To determine whether tri/tetra-antennary complex *N*–glycan surface expression of human Treg also correlates with suppressive potency, activated cocultures were prepared using FACS-purified PHA-L^high^ and PHA-L^low^ human Treg and naïve- and memory-phenotype CD4^+^ Tconv (Figure 11B). In these assays, PBMC-derived Treg suppressed the proliferation of naïve but not the memory CD4^+^ T-conv subset. The PHA-L^high^ Treg demonstrated higher suppressive potency for naïve CD4^+^ Tconv than their PHA-L^low^ counterparts (Figures 11C–F). Taken together, the findings for mouse and human indicate that binding intensity of PHA-L to surface *N*-glycans identifies Treg with differential suppressive potency.

## Discussion

In this study, we report a comprehensive lectin-based characterization of the Treg surface glycome and its relationship with Treg phenotype and suppressive function. In both mouse and human, distinct differences were observed between the surface lectin-binding profiles of Treg and CD4^+^ Tconv. In mouse, surface glycosylation was found to vary among Treg from different immunological niches and Treg activation resulted in surface glycome remodeling. The surface density of tri/tetra-antennary complex *N*-glycans (based on lectin-binding patterns) correlated with expression of proteins linked to Treg identity and effector functions and Treg with higher surface density of these *N*-glycan structures displayed greater suppressive potency. Furthermore, partial stripping of surface tri/tetra-antennary complex *N*-glycans resulted in a significant loss of Treg capacity to suppress early pro-proliferative activation events in Tconv. Therefore, the variances in surface glycosylation described here reveal a novel form of Treg heterogeneity that is relevant to their localization, activation status and suppressive functions.

### Distinctive Surface Glycosylation of Freshly Isolated Treg and Tconv

The finding that freshly isolated human and mouse-derived Treg display different levels of expression of multiple glycan epitopes when compared to Tconv has not been previously reported and represents a new distinguishing feature of Treg. However, it is important to note that none of the lectins tested showed exclusive binding to either of the two T-cell subtypes studied. Thus, it can be concluded that both regulatory and non-regulatory T-cells express the range of different glycan structures that were identified through lectin staining but do so, in many cases, at distinctly different levels. Although lectin exclusivity for binding to Treg would be desirable for the development of lectin-based Treg purification methods, its absence is not surprising given the known complexity and diversity of surface glycosylation in mammalian lymphocytes and other cells (42). Mass spectrometry or other direct analysis of carbohydrates released from the surfaces of Treg and Tconv will be necessary for a full understanding of how their surface glycans differ under physiological conditions.

### Surface Glycosylation Differences among Treg from Different Anatomical Locations

T-cell development, differentiation, and activation are accompanied by changes in gene expression including differential regulation of enzymes involved in glycosylation pathways. These gene regulatory events ultimately lead to altered glycosylation characteristics across the various stages of the T-cell life-cycle (17–19, 43–45). Thus, the glycosylation differences observed in T-cells from different anatomical locations in mice may reflect the presence of T-cell subsets in different developmental stages and activation states at the various locations. For example, peanut agglutinin lectin (PNA) surface binding was found to be substantially higher on both Treg and CD4^+^ Tconv from the thymus and bone marrow than it was on the equivalent cells from secondary lymphoid organs (see Table S1 in Supplementary Material). This is in accordance with previous studies that have reported high expression of core 1 structures by developing T-cells and by T-cells that have acquired a memory phenotype (17, 46).

Variability of T-cells across different anatomical sites has also been observed during the study of the expression of lymphoid tissue homing receptors by different regulatory and non-regulatory T-cell subsets (47, 48). Moreover, the expression of the same homing receptor has been shown to be differentially expressed by Treg and Tconv isolated from the same anatomical site, both in the resting state and after immunization (47, 49). In addition, Treg from mouse and human have been shown to differ from Tconv in their efficiency to upregulate memory-, inflammation-, and non-lymphoid tissue-related trafficking receptors (47, 50). Notably, glycosylation is also known to mediate T-cell trafficking and homing to the lymphoid organs and to modulate T-cell effector function (14, 15, 17). Therefore, the further pursuit of this observation should attempt to determine how the observed glycosylation variability across the different anatomical sites relates to *in vivo* Treg migratory behavior and location-specific functional characteristics.

Interestingly, the differences in the binding of multiple lectins to Treg and Tconv that were detected in multiple anatomical niches in the mouse, were predominantly absent or diminished in peripheral blood-derived T-cells. Similarly, the differences in lectin binding to human Treg and Tconv circulating in the blood were not as clear. Given that Treg suppressive functions correlated with glycan expression levels, it will be of interest to learn whether human Treg from other anatomical sites display more distinctive surface glycan profiles and suppressive abilities compared to PBMC-derived Treg.

### Activation-Induced Remodeling of the Treg and Tconv Surface Glycome

Changes that occur to the surface glycan landscape of T-cells subtypes are likely to represent an important aspect of antigen-specific immune regulation. For example, activation through TCR signaling promotes *N*-glycan branching by upregulation of α-mannosidases and *Mgat5* at the transcriptional level, while maintaining low levels of *Mgat1* and *Mgat2*, which compete for the same sugar substrates (51). This previously reported activation-induced upregulation of *N*-glycan expression is in accordance with the observed higher PHA-L binding to previously activated (CD62L^lo^) Treg *in vivo* as well as to Treg which were activated *in vitro*. Furthermore, the higher PHA-L binding to Treg when compared to Tconv that was observed in the resting state lends support to a previously posited model whereby Treg are proposed to maintain a semi-activated phenotype during health (52, 53). Given that glycosylation has been reported to influence T-cell function and that other observations described in this work suggest an important role for surface glycosylation in modulating Treg suppressive functions, it will now be of interest to further explore the mechanisms underlying activation-induced surface glycosylation changes in Treg and its relevance to Treg-based immunotherapies.

### Treg Surface Glycosylation As a Marker of Suppressive Capacity

A novel observation from our results is that greater binding of PHA-L and scFv-A4 correlated with higher expression levels of various markers that are known to be linked with Treg identity and/or suppressive potency. Recently, Wyss et al. reported the utility of differential GITR, PD-1 and CD25 expression for the identification of Treg populations that differ in their TCR repertoires and regulatory functions. High expression of these markers was shown to identify a highly self-reactive Treg subset responsible for the control of lymphocyte proliferation in the peripheral lymph nodes. Conversely, Treg expressing low levels of GITR, PD-1 and CD25 were less self-reactive but were involved in prevention of colitis (54). Since Treg GITR and PD-1 expression levels were associated with the binding intensities of multiple lectins in the current study, differential glycan expression may represent an alternative approach for discrimination between Treg subsets with distinct regulatory properties.

In eukaryotic cells, *N*-glycosylation plays a crucial role in mediating proper protein folding and quality control in the endoplasmic reticulum as well as intracellular trafficking to the Golgi apparatus (GA), where carbohydrate structures undergo further processing (55–57). While potential protein glycosylation sites are encoded in the genome, the carbohydrate structures that are produced and attached to such sites depend on enzymatic activities in the GA and on the metabolic availability of their substrates (58–60). The resulting number and degree of branching of *N*-glycans determine protein binding to galectins and formation of the galectin–glycoprotein lattice at the cell surface. This lattice regulates key cellular functions, such as endocytosis and localization, mobility, and clustering of proteins within the plasma membrane (58, 61). The correlations we observed between surface *N*-glycan expression and Treg functional markers may, thus, reflect a direct relationship between the activity of posttranslational glycosylation pathways and the translocation and stability of glycoproteins at the cell surface. In keeping with a functional significance for this relationship, purification of mouse Treg subpopulations using the lectin PHA-L confirmed that surface levels of tri/tetra-antennary complex *N-*glycans correlate with enhanced ability to suppress CD4^+^ and CD8^+^ T-cell proliferation.

Given that we observed higher PHA-L surface binding to CD62L^low^ compared to CD62L^high^ Treg, it could be postulated that the greater suppressive potency observed for purified PHA-L^high^ Treg simply reflects an expected functional difference between memory- and naïve-phenotype cells. However, in other analyses, we have observed that PHA-L^high^ and PHA-L^low^ Treg each contain substantial proportions of CD62L^low^ cells and that purified CD62L^high^ and CD62L^low^ Treg have equal suppressive potency against CD4^+^ responder Tconv in the coculture assay system we have employed (data not shown). Thus, while purified, highly *N*-glycosylated Treg are likely to contain a greater proportion of memory-phenotype Treg than those with lower surface *N*-glycan levels, this does not seem to account for their higher suppressive potency. Although less evident than in the mouse, purified human PHA-L^high^ Treg also tended to be more suppressive of naïve (but not memory) CD4^+^ T-cell proliferation than their PHA-L^low^ counterparts. These observations demonstrate, for the first time, that differential surface glycan expression may be exploited to identify T-cell subpopulations with variable functional potency.

### Functional Effects of Manipulation of Treg Surface *N*-Glycosylation

Abnormal *N*-glycan processing, particularly *Mgat5* deficiency, has been associated with the development of autoimmunity due to effector T-cell hyper-activity (62, 63). The expression of α-(1,2)-mannosidase has also been identified as a marker for induction and maintenance of immunological tolerance *in vivo*. Sawitzki et al. showed that upregulation of α-(1,2)-mannosidase is associated with allograft acceptance in rat and mouse (64). Furthermore, in a subsequent study, allo-antigen activation of mouse Treg led to upregulation of α-(1,2)-mannosidase expression and alterations to surface protein *N*-glycosylation (15). Inhibition of α-(1,2)-mannosidase (preventing *N*-glycan processing) resulted in impaired Treg adhesive properties and migration to allograft-draining lymph nodes but did not influence Treg suppressive potency (22). In contrast, we have found that partial removal of *N*-glycan structures (through enzymatic digestion) resulted in clear loss of Treg capacity for *in vitro* suppression of Tconv. Experiments involving enzymatic or genetic manipulation to individual terminal glycan moieties could provide more refined detail regarding the functional significance of the various lectin-binding differences between Treg and Tconv. However, successful application of such approaches in purified primary lymphocytes will present significant technical challenges.

Although the specific molecular mechanism for the loss of Treg suppressive potency following PNGase F treatment remains to be elucidated, we have observed that it is contact-dependent in the assay system used in our experiments and does not require the presence of an APC. Moreover, despite the importance of *N*-glycosylation for many ligand/receptor interactions, the loss of suppressive capacity was not associated with impaired ability of the manipulated Treg to form stable conjugates with DC or Tconv. As PNGase F-treated Treg upregulated activation-associated molecules, such as CD25, CD69, and PD-1, to the same or even greater extent than non-treated Treg following TCR stimulation, the effects of partial removal of surface *N-*glycans on Treg suppression do not reflect a broad state of unresponsiveness.

Interestingly, we have observed that PNGase F treatment of naïve CD4^+^ and CD8^+^ Tconv results in a moderately higher rate of CD69 and CD25 upregulation following 24 h of activation with minimal effect on the subsequent rate of proliferation (data not shown). Thus, temporary modulation of the surface *N*-glycan profile does not broadly prevent the activation-induced effector functions of all primary T-cells. Since many surface proteins were likely to have been affected by the enzymatic manipulation, we hypothesize that the observed loss of Treg suppressive potency reflects compromised function of multiple contact-dependent accessory pathways operating at the Treg/Tconv interface. Among the pathways described as mediating the direct suppression of Tconv activation by Treg in the context of cell-cell contact are the localized release or generation of soluble mediators such as IL-35, galectin 1, cAMP, adenosine and perforin/granzymes (4, 65) as well as surface-bound glycoproteins such as PD-1, PD-L1 and CD69 (66, 67). Whether one or more of these individual mediators is specifically vulnerable to removal of *N*-glycans will require further investigation.

In conclusion, the results described here indicate that glycosylation plays a crucial and distinctive role in Treg biology. Further molecular and mechanistic investigation of this role should provide novel insights of relevance to human immunological disease and immunotherapy.

## Ethics Statement

Human peripheral blood sampling from healthy adult volunteers was carried out in accordance with the recommendations of the Policies and Procedures of the Research Ethics Committee of the National University of Ireland, Galway. All subjects gave written informed consent in accordance with the Declaration of Helsinki. The protocol was approved by the Research Ethics Committee of the National University of Ireland, Galway. Breeding, housing, and euthanasia of FOXP3-EGFP C57BL/6 mice was carried out in accordance with the recommendations of the Policies and Procedures of the National University of Ireland, Galway Animal Care Research Ethics Committee (ACREC). The protocol was approved by ACREC and was carried out under individual authorization from the Health Products Regulatory Authority of Ireland and the Environmental Protection Agency.

## Author Contributions

JC was responsible for the conception of the work, experimental design, acquisition, analysis and interpretation of the data, writing, and final approval of the manuscript. SH, JG, and SC contributed to experimental design, data analysis and interpretation, and writing and final approval of the manuscript. NO designed and performed all statistical analyses and participated in the writing and final approval of the manuscript. TR, RC, and LJ contributed to the conception of the work, interpretation of the data, and the writing and final approval of the manuscript. MG was responsible for the conception, funding, and supervision of the work, contributed to the experimental design, interpretation of the data, writing, and final approval of the manuscript.

## Conflict of Interest Statement

The authors declare that the research was conducted in the absence of any commercial or financial relationships that could be construed as a potential conflict of interest.
