# Supplementary figures and images for: Distinctive Surface Glycosylation Patterns Associated With Mouse and Human CD4+ Regulatory T Cells and Their Suppressive Function
Source: Front Immunol. 2017 Aug 21;8:987. doi: 10.3389/fimmu.2017.00987 (PMC5566562; doi:10.3389/fimmu.2017.00987)

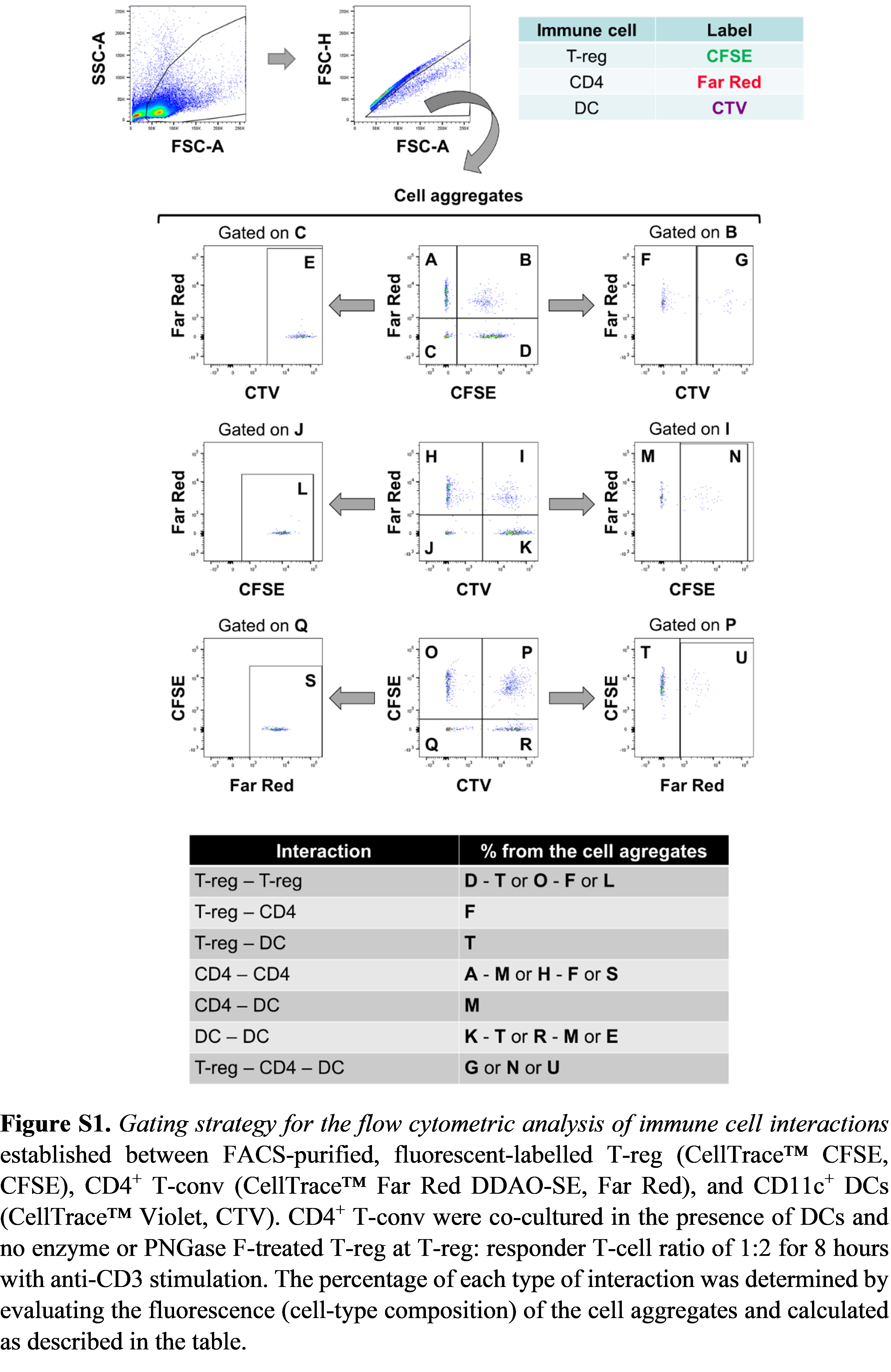

Supplement: Supplementary file 1 [file image_1.tif]
