# Supplementary material for: Distinctive Surface Glycosylation Patterns Associated With Mouse and Human CD4+ Regulatory T Cells and Their Suppressive Function
Source: Front Immunol. 2017 Aug 21;8:987. doi: 10.3389/fimmu.2017.00987 (PMC5566562; doi:10.3389/fimmu.2017.00987)
Supplement: Supplementary file 2 [file table_1.docx]

**Table S1.** Summary of results of flow cytometric screening of lectin binding to T-reg and T-conv from healthy mouse spleen, subcutaneous lymph nodes, mesenteric lymph nodes, thymus, bone marrow and PBLs^*^

| **Lectin** | **Major ligand(s)** | **Spleen** | | | **Subcutaneous lymph nodes** | | | **Mesenteric lymph nodes** | | |
| --- | --- | --- | --- | --- | --- | --- | --- | --- | --- | --- |
|  |  | **T-conv** | **T-reg** | **p value**† | **T-conv** | **T-reg** | **p value**† | **T-conv** | **T-reg** | **p value**† |
| **AAA** | **α-Fuc** | 119.3 ± 37.91 | 114.7 ± 39.80 | 0.5 | 99.56 ± 53.81 | 95.74 ± 58.55 | 0.438 | 80.88 ± 6.243 | 73.24 ± 15.91 | 0.375 |
| **AAL** | **Fuc-α-(1,6)-** | **39240 ± 27420** | **41880 ± 26280** | **0.0312** | **39980 ± 23590** | **42830 ± 22650** | **0.0156** | 30710 ± 9222 | 31210 ± 9772 | 0.492 |
| **UEA-I** | **Fuc-α-(1,2)-** | 118 ± 47.38 | 112.7 ± 30.79 | 0.688 | 95.76 ± 44.17 | 91.56 ± 38.54 | 0.5 | **86.02 ± 6.370** | **71.18 ± 11.00** | **0.0625** |
| **MAL-II** | **Sialic acid-α-(2,3)-Gal** | 24480 ± 7340 | 23920 ± 5484 | 0.734 | **27390 ± 9244** | **23770 ± 8278** | **0.00781** | **24250 ± 9175** | **22810 ± 8130** | **0.0234** |
| **SNA-I** | **Sialic acid-α-(2,6)-Gal/GalNAc** | **8103 ± 2232** | **11440 ± 3420** | **0.00781** | **7035 ± 2140** | **9587 ± 2960** | **0.00781** | **6777 ± 2458** | **8925 ± 3251** | **0.00781** |
| **ConA** | **Man** | 8485 ± 3302 | 7687 ± 2427 | 0.117 | 6890 ± 1985 | 7113 ± 2128 | 0.125 | **6509 ± 3159** | **6992 ± 3575** | **0.00781** |
| **GNL** | **Man-α-(1,3)-** | **10490 ± 5006** | **9230 ± 4769** | **0.00781** | 11100 ± 5237 | 10790 ± 5105 | 0.109 | **9551 ± 2899** | **9180 ± 2923** | **0.0234** |
| **NPL** | **Man-α-(1,6)-** | 5512 ± 3214 | 5093 ± 3372 | 0.266 | 5417 ± 2862 | 5601 ± 3138 | 0.234 | 4104 ± 1115 | 4176 ± 1145 | 0.289 |
| **PSA** | **Man (Fuc-dependent)** | **57580 ± 12140** | **67230 ± 13450** | **0.00781** | **52300 ± 6627** | **63840 ± 9781** | **0.00781** | **57690 ± 14760** | **67150 ± 15870** | **0.00781** |
| **GSL-I** | **α-Gal/ α-GalNAc** | **793.8 ± 553.7** | **4076 ± 2163** | **0.00781** | **315.4 ± 190.9** | **2777 ± 2089** | **0.00781** | **366 ± 128.4** | **2465 ± 702.8** | **0.00781** |
| **Jacalin** | **Gal (sialylation tolerant)** | **1828 ± 795.2** | **1289 ± 477.4** | **0.00781** | **1543 ± 675.0** | **1077 ± 440.1** | **0.00781** | **1568 ± 659.4** | **1185 ± 489.7** | **0.00781** |
| **PNA** | **Gal-β-(1,3)-GalNAc** | 398.3 ± 228.1 | 456.5 ± 112.4 | 0.352 | **278.3 ± 136.3** | **398.3 ± 136.9** | **0.00781** | **258.6 ± 129.6** | **369.4 ± 143.0** | **0.00781** |
| **SJA** | **β-GalNAc** | 124.4 ± 30.35 | 116.2 ± 31.34 | 0.125 | 91.52 ± 17.91 | 82.62 ± 20.58 | 0.250 | **113.5 ± 26.80** | **97.44 ± 26.24** | **0.0625** |
| **DSL** | **GlcNAc** | **1131 ± 837.8** | **2532 ± 1859** | **0.00781** | **859.5 ± 739.4** | **2556 ± 1877** | **0.00781** | **878.8 ± 674.6** | **2385 ± 1681** | **0.00781** |
| **PHA-E** | **Biantennary, bisecting**  **GlcNAc,β-Gal/Gal-(1,4)GlcNAc** | **3297 ± 1024** | **11080 ± 1979** | **0.00781** | **2508 ± 1083** | **8868 ± 2436** | **0.00781** | **3281 ± 1437** | **10220 ± 3486** | **0.00781** |
| **PHA-L** | **Tri-/tetra-antennary**  **β-Gal/Gal-β-(1,4)-GlcNAc** | **2418 ± 2170** | **10720 ± 7480** | **0.00781** | **1578 ± 1749** | **8193 ± 6805** | **0.00781** | **1238 ± 1076** | **6268 ± 3932** | **0.00781** |
| **RCA-I** | **Gal-β-(1,4)-GlcNAc** | **25470 ± 8410** | **32190 ± 13250** | **0.0234** | **25240 ± 5776** | **32010 ± 8086** | **0.00781** | **1239 ± 1076** | **34920 ± 11830** | **0.00781** |

| **Lectin** | **Major ligand(s)** | **Thymus** | | | **Bone marrow** | | | **PBLs** | | |
| --- | --- | --- | --- | --- | --- | --- | --- | --- | --- | --- |
|  |  | **T-conv** | **T-reg** | **p value**† | **T-conv** | **T-reg** | **p value**† | **T-conv** | **T-reg** | **p value**† |
| **AAA** | **α-Fuc** | 67.42 ± 10.54 | 63.76 ± 31.49 | 0.812 | 459.8 ± 302.7 | 358.0 ± 246.2 | 0.125 | 56.02 ± 4.333 | 37.28 ± 15.88 | 0.125 |
| **AAL** | **Fuc-α-(1,6)-** | **44430 ± 7963** | **45970 ± 8023** | **0.0781** | 42340 ± 8793 | 43500 ± 6293 | 0.750 | **19560 ± 5610** | **16820 ± 5716** | **0.0625** |
| **UEA-I** | **Fuc-α-(1,2)-** | 84.46 ± 17.31 | 96.12 ± 15.69 | 0.4237 | 328.5 ± 101.1 | 261.3 ± 79.19 | 0.125 | 55.48 ± 1.827 | 48.36 ± 5.704 | 0.125 |
| **MAL-II** | **Sialic acid-α-(2,3)-Gal** | **14850 ± 3683** | **28820 ± 8197** | **0.00781** | 19920 ± 5395 | 19990 ± 5675 | 1.000 | **17750 ± 8531** | **14080 ± 7292** | **0.0625** |
| **SNA-I** | **Sialic acid-α-(2,6)-Gal/GalNAc** | **9547 ± 3013** | **11280 ± 3156** | **0.00781** | **8779 ± 3462** | **13570 ± 5217** | **0.0156** | **3655 ± 828.7** | **4643 ± 1298** | **0.0625** |
| **ConA** | **Man** | **8750 ± 2534** | **7033 ± 1950** | **0.00781** | 7742 ± 1981 | 7352 ± 2309 | 0.266 | 3353 ± 1530 | 3344 ± 1644 | 0.938 |
| **GNL** | **Man-α-(1,3)-** | **17860 ± 7294** | **9112 ± 2927** | **0.0156** | **12960 ± 5416** | **8424 ± 1386** | **0.0938** | **5657 ± 1391** | **4840 ± 1480** | **0.0625** |
| **NPL** | **Man-α-(1,6)-** | **13130 ± 5851** | **5900 ± 3861** | **0.00781** | **10840 ± 8900** | **4277 ± 523.0** | **0.0625** | **2121 ± 335.5** | **1854 ± 339.4** | **0.0625** |
| **PSA** | **Man (Fuc-dependent)** | **64150 ± 12260** | **73910 ± 14490** | **0.00781** | **80040 ± 12000** | **98060 ± 12400** | **0.0156** | 31940 ± 13340 | 33820 ± 16870 | 0.375 |
| **GSL-I** | **α-Gal/ α-GalNAc** | **2794 ± 1039** | **6242 ± 4267** | **0.00781** | 5223 ± 7569 | 5132 ± 945.6 | 1.000 | **127.2 ± 26.71** | **472.2 ± 165.7** | **0.0625** |
| **Jacalin** | **Gal (sialylation tolerant)** | **2769 ± 1038** | **1031 ± 415.2** | **0.00781** | **1809 ± 1086** | **1028 ± 455.7** | **0.0156** | **929.6 ± 174.9** | **478.6 ± 78.32** | **0.0625** |
| **PNA** | **Gal-β-(1,3)-GalNAc** | **2457 ± 571.8** | **904.5 ± 243.4** | **0.00781** | 1008 ± 663.3 | 752.7 ± 134.0 | 0.359 | 198 ± 25.01 | 179.2 ± 21.29 | 0.0625 |
| **SJA** | **β-GalNAc** | 84.6 ± 28.34 | 89.74 ± 47.32 | 0.750 | 386.3 ± 147.9 | 269.8 ± 68.72 | 0.125 | **60.88 ± 11.48** | **39.68 ± 8.722** | **0.0625** |
| **DSL** | **GlcNAc** | 2740 ± 1747 | 2628 ± 1684 | 0.180 | 1852 ± 2036 | 1356 ± 541.1 | 0.703 | **230 ± 65.30** | **411.8 ± 194.9** | **0.0625** |
| **PHA-E** | **Biantennary, bisecting**  **GlcNAc,β-Gal/Gal-(1,4)GlcNAc** | 14290 ± 1714 | 14390 ± 2378 | 0.836 | 9808 ± 6676 | 10770 ± 2607 | 0.594 | **791.6 ± 321.7** | **2432 ± 1272** | **0.0625** |
| **PHA-L** | **Tri-/tetra-antennary**  **β-Gal/Gal-β-(1,4)-GlcNAc** | **9928 ± 3047** | **11090 ± 3697** | **0.0391** | **4856 ± 2962** | **8131 ± 2373** | **0.0625** | **421 ± 98.66** | **1682 ± 816.8** | **0.0625** |
| **RCA-I** | **Gal-β-(1,4)-GlcNAc** | 54200 ± 19660 | 56520 ± 18160 | 0.578 | **37740 ± 8898** | **69390 ± 25060** | **0.0156** | 24260 ± 9410 | 23730 ± 10190 | 0.500 |

^*^Surface glycosylation was evaluated using lectin profiling by flow cytometry. Single cell suspensions were obtained from spleen, subcutaneous lymph nodes, mesenteric lymph nodes (Upper Table), thymus, bone marrow and PBLs (Lower Table) from n=4-8 C57BL/6 FoxP3.EGFP mice. Mean values for median fluorescence intensity ± SD are shown for both populations. † Statistical analysis was performed by permutation test with a paired design.
