# Supplementary material for: Distinctive Surface Glycosylation Patterns Associated With Mouse and Human CD4+ Regulatory T Cells and Their Suppressive Function
Source: Front Immunol. 2017 Aug 21;8:987. doi: 10.3389/fimmu.2017.00987 (PMC5566562; doi:10.3389/fimmu.2017.00987)
Supplement: Supplementary file 3 [file table_2.docx]

**Table S2.** Lectins used for glycoanalysis.

| Abbreviation | Origin | Species | Common name | Major Ligand(s) | (µg/ml)^*^ | Catalogue number | Supplier |
| --- | --- | --- | --- | --- | --- | --- | --- |
| Jacalin | plant | *Artocarpus integrifolia* | Jack fruit lectin | Gal (sialylation tolerant) | 0.125 | B-1155 | Vector Labs |
| SJA | plant | *Sophora japonica* | Pagoda tree lectin | β-GalNAc | 1 | B-1135 | Vector Labs |
| PNA | plant | *Arachis hypogaea* | Peanut lectin | Gal-β(1,3)-GalNAc | 1 | B-1075 | Vector Labs |
| DSL | plant | *Datura stramonium* | Jimson weed lectin | GlcNAc | 0.4 | B-1185 | Vector Labs |
| NPL | plant | *Narcissus pseudonarcissus* | Daffodil lectin | Man-α(1,6)- | 2.5 | B-1375 | Vector Labs |
| GNL | plant | *Galanthus nivalis* | Snowdrop lectin | Man-α(1,3)- | 7.5 | B-1245 | Vector Labs |
| ConA | plant | *Canavalia ensiformis* | Jack bean lectin | Man | 1 | B-1005 | Vector Labs |
| PSA | plant | *Pisum sativum* | Pea lectin | Man (Fuc-dependent) | 1 | B-1055 | Vector Labs |
| MAL-II | plant | *Maackia amurensis* | Maackia agglutinin II | Sialic acid-α(2,3)-Gal | 0.25 | B-1265 | Vector Labs |
| SNA-I | plant | *Sambucus nigra* | Sambucus lectin-I | Sialic acid-α(2,6)-Gal/GalNAc | 2 | BA-6802-1 | EY Labs |
| PHA-L | plant | *Phaseolus vulgaris* | Kidney bean leukoagglutinin | Tri/tetraantennary  βGal/Gal-β(1,4)-GlcNAc | 0.5 | B-1115 | Vector Labs |
| PHA-E | plant | *Phaseolus vulgaris* | Kidney bean erythroagglutinin | Biantennary, bisecting  GlcNAc,βGal/Gal-β(1,4)GlcNAc | 1 | B-1125 | Vector Labs |
| RCA-I | plant | *Ricinus communis* | Castor bean lectin I | Gal-β(1,4)-GlcNAc | 1 | B-1085 | Vector Labs |
| AAL | fungi | *Aleuria aurantia* | Orange peel fungus lectin | Fuc-α(1,6)- | 1 | B-1395 | Vector Labs |
| AAA | animal | *Anguilla anguilla* | European Eel Lectin | α-Fuc | 5 | BA-4901-1 | EY Labs |
| UEA-I | plant | *Ulex europaeus* | Gorse lectin-I | Fuc-α(1,2)- | 1 | B-1065 | Vector Labs |
| GSL-I | plant | *Griffonia simplicifolia*  *(Bandeiraea simplicifolia)* | Griffonia/ Bandeiraea Lectin I | α-Gal/ α-GalNAc | 1 | B-1105 | Vector Labs |

^*^concentration of lectin used to stain 10^6^ cells
