# Supplementary material for: Distinctive Surface Glycosylation Patterns Associated With Mouse and Human CD4+ Regulatory T Cells and Their Suppressive Function
Source: Front Immunol. 2017 Aug 21;8:987. doi: 10.3389/fimmu.2017.00987 (PMC5566562; doi:10.3389/fimmu.2017.00987)
Supplement: Supplementary file 4 [file table_3.docx]

**Table S3.** Antibodies used for flow cytometry.

| Antibody | Species/ Isotype | Clone | Supplier |
| --- | --- | --- | --- |
|  |  |  |  |
| Purified |  |  |  |
| Anti-mouse CD3ε | Armenian Hamster IgG | 145-2C11 | eBioscience |
| Anti-mouse CD28 | Golden Syrian Hamster IgG | 37.51 | eBioscience |
| FITC-Conjugated |  |  |  |
| Anti-human CD4 | Mouse IgG1, κ | RPA-T4 | eBioscience |
| PE-Conjugated |  |  |  |
| Anti-mouse CD62L | Rat IgG2a, κ | MEL-14 | eBioscience |
| Anti-mouse CD69 | Armenian Hamster IgG | H1.2F3 | eBioscience |
| Anti-human CD127 | Mouse IgG1, κ | eBioRDR5 | eBioscience |
| Anti-mouse CD73 | Rat Wistar IgG2a, κ | TY/23 | BD Biosciences |
| Anti-mouse CD103 | Armenian Hamster IgG | 2E7 | eBioscience |
| Anti-mouse CD152 (CTLA-4) | Armenian Hamster IgG | UC10-4B9 | eBioscience |
| Anti-mouse CD274 (PD-L1) | Rat IgG2a, λ | MIH5 | eBioscience |
| Anti-mouse CD278 (ICOS) | Rat IgG2b, κ | 7E.17G9 | eBioscience |
| Anti-mouse CD279 (PD-1) | Rat IgG2b, κ | RMP1-30 | eBioscience |
| Anti-mouse CD357 (GITR) | Rat IgG2b, κ | DTA-1 | eBioscience |
| Anti-mouse Helios | Armenian Hamster IgG | 22F6 | eBioscience |
| PE.Cy7-Conjugated |  |  |  |
| Anti-mouse CD62L | Rat IgG2a, κ | MEL-14 | eBioscience |
| PerCP-eFluor710-Conjugated |  |  |  |
| Anti-mouse LAP | Mouse IgG1, κ | TW7-16B4 | eBioscience |
| APC-Conjugated |  |  |  |
| Anti-mouse CD4 | Rat IgG2a, κ | RM4-5 | eBioscience |
| Anti-mouse CD8α | Rat IgG2a, κ | 53-6.7 | eBioscience |
| Anti-human CD45RA | Mouse IgG2b, κ | HI100 | eBioscience |
| APC-eFluor780-Conjugated |  |  |  |
| Anti-mouse CD8α | Rat IgG2a, κ | 53-6.7 | eBioscience |
| Anti-mouse TCR-β | Armenian Hamster IgG | H57-597 | eBioscience |
| eFluor450-Conjugated |  |  |  |
| Anti-mouse CD11c | Armenian Hamster IgG | N418 | eBioscience |
| Anti-mouse CD25 | Rat IgG1, λ | PC61.5 | eBioscience |
| Anti-mouse GARP | Rat IgG2a, κ | YGIC86 | eBioscience |
| Biotin-Conjugated |  |  |  |
| anti-HA | mouse IgG1 | GG8-1F3.3.1 | Miltenyi Biotec |
